# Convergence and Properties of Intrinsic Bond Orbitals in Solids

**DOI:** 10.1021/acs.jctc.5c00130

**Published:** 2025-10-16

**Authors:** Benjamin Wöckinger, Alexander Rumpf, Tobias Schäfer

**Affiliations:** Institute for Theoretical Physics, 27259 TU Wien, Wiedner Hauptstraße 8-10/136, Vienna A-1040, Austria

## Abstract

We present a study of the construction and spatial properties of localized Wannier orbitals in large supercells of insulating solids using plane waves as the underlying basis. The Pipek-Mezey (PM) functional in combination with intrinsic atomic orbitals (IAOs) as projectors is employed, resulting in so-called intrinsic bond orbitals (IBOs). Independent of the bonding type and band gap, a correlation between orbital spreads and geometric properties is observed. As a result, comparable sparsity patterns of the Hartree–Fock exchange matrix are found across all considered bulk 3D materials, exhibiting covalent bonds, polar covalent bonds, and ionic bonds. Recognizing the considerable computational effort required to construct localized Wannier orbitals for large periodic simulation cells, we address the performance and scaling of different solvers for the localization problem. This includes the Broyden–Fletcher–Goldfarb–Shanno (BFGS), Conjugate-Gradient (CG), Steepest Ascent (SA), as well as the Direct Inver-sion in the Iterative Subspace (DIIS) method. Each algorithm performs a Riemannian optimization under a unitary matrix constraint, efficiently reaching the optimum in the “curved parameter space” on geodesics. The solvers have been implemented both within the VASP and as a standalone open-source software package. Furthermore, we observe that the construction of Wannier orbitals for supercells of metal oxides presents a significant challenge, requiring approximately 1 order of magnitude more iteration steps than other systems studied.

## Graphical abstract

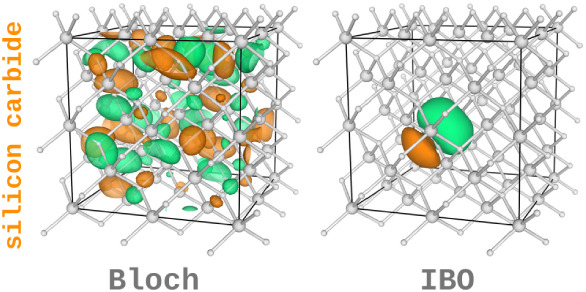

## 1 Introduction

Localized orbitals are a useful tool in quantum chemistry and materials physics. They serve a variety of purposes, for example, the analysis of chemical bonds in tune with chemical intuition,^1,2^ the investigation of electron transfer processes,^3^ the calculation of electron–phonon interactions,^4^ or the development of efficient many-electron correlation algorithms by introducing sparsity in electron repulsion integrals.^5–11^

Known as localized Wannier orbitals in solid-state physics and localized molecular orbitals in quantum chemistry, they are usually derived from delocalized one-electron mean-field orbitals through rotations, achieved by a unitary matrix, resulting in spatial confinement. Various definitions have been proposed for determining this unitary matrix, with several implementations available for periodic systems. Spatial confinement can be achieved by minimizing the orbital spread, a technique known as Foster-Boys (FB) localization.^12^ Alternatively, maximizing electronic self-repulsion, termed Edmiston-Ruedenberg (ER) localization^1^ or maximizing self-overlap, known as von-Niessen (VN) localization^13^ can be employed. Another approach, Pipek-Mezey (PM) localization^14^ utilizes atomic partial charges as the localization measure. While these methods require iterative optimization, single-shot localization techniques also exist for solids.^4,8,15^

Early implementations for periodic boundary conditions primarily focused on the FB localization scheme, with applications for plane-wave basis sets^16,17^ and atom-centered basis functions.^18^ While Riemannian optimization strategies for determining the optimal unitary transformation matrix were applied to molecules by Lehtola et al.,^19^ the PM localization technique was adapted to periodic systems using a Riemannian optimization approach by Jónsson et al.^20^ They introduced the notion of generalized PM Wannier orbitals by employing various partial charge estimates for the PM functional. Building on the work by Jónsson et al., subsequent implementations of generalized PM Wannier orbitals for solids were reported.^9,21–23^ The intrinsic bond orbitals (IBOs) discussed in this work can similarly be understood as a form of generalized PM Wannier functions.

A central challenge in constructing these localized Wannier orbitals lies in efficiently determining the optimal unitary transformation. This process can be considered as a Riemannian optimization problem under unitary constraints, but the performance of different algorithms within this framework is not fully clear. Previous work, such as that by Clement et al.^21^ suggested the superiority of the limited-memory BFGS (L-BFGS) over the Conjugate-Gradient (CG) solver. Our investigations within the Riemannian optimization context, however, reveal a different picture, demonstrating that both solvers exhibit comparable performance. We attribute this discrepancy to fundamental differences in the computational setup compared to Clement et al.’s approach, which we discuss within our study.

Furthermore, the scalability of Wannier orbital construction with respect to system size remains a significant challenge, especially for applications targeting realistic models of surfaces and defects, which necessitate large simulation cells. We address the critical question of how the number of iterations required for convergence scales with the number of atoms, providing crucial insights for the application of localized orbitals to increasingly complex materials. Additionally, we assess whether the Direct Inversion in the Iterative Subspace (DIIS) technique^24,25^ can accelerate the convergence of the iterative optimization.

Finally, a key objective of our work is to leverage localized orbitals to introduce sparsity into Coulomb integrals, aiming to mitigate the computational bottleneck of wave function based methods. A prevailing concern has been the potential impact of small band gaps on the sparsity of electron repulsion integrals, which could hinder the effectiveness of local correlation approaches. Here we investigate the sparsity of the Fock exchange matrix and demonstrate that, for the semiconductors considered, the sparsity is remarkably robust and largely unaffected by the band gap.

The paper is divided into two main parts. The first main part starts with Section 2 and discusses the theory, implementation, and performance of different numerical solvers to numerically construct IBOs. The second main part starts with Section 6 where we report spatial properties of IBOs, an analysis of the sparsity of the Fock exchange matrix, and trends across the considered materials.

## 2 Theory

### 2.1 Intrinsic Bond Orbitals

In solids, intrinsic bond orbitals (IBOs), $\left|W_{Rj}\right\rangle$ , can be defined as generalized Wannier orbitals.^9,17^ They are constructed as superpositions of Bloch orbitals, $\left|\chi_{jk}\right\rangle$ , obtained from prior mean-field calculations such as Hartree-Fock (HF) or Kohn–Sham density functional theory (DFT),

1 $$\left|W_{Rj}\right\rangle =\frac{1}{V_{\mathrm{BZ}}}\int_{\mathrm{BZ}}d^{3}ke^{-ikR}\overset{N_{\mathrm{occ}}}{\underset{i}{\sum }}u_{ij}^{\left(k\right)}\left|\chi_{jk}\right\rangle$$

Here, $u_{ij}^{\left(k\right)}$ is a unitary matrix at each k-point ***k***, and *V*_BZ_ represents the volume of the Brillouin zone (BZ).

In this work, all calculations are based on (HF) orbitals obtained from the plane-wave based VASP.^27–29^ Supercells are considered using a **Γ**-only sampling of the BZ, reducing eq 1 to

2 $$\left|W_{j}\right\rangle =\overset{N_{\mathrm{occ}}}{\underset{i}{\sum }}u_{ij}\left|\chi_{j}\right\rangle$$

The matrix *u*_*ij*_ is optimized to maximize (minimize) a localization functional L , which defines the localized Wannier orbitals. Various localization functionals exist in the literature, such as FB^12^ (ER)^1^ (VN)^13^ and Pipek-Mezey (PM).^14^ Our Riemannian optimization^30^ algorithm described in Section 2.2.2 is suited for any cost functional, allowing us to compare the case of PM, FB, and VN. Since we employ **Γ**-point-only sampling, the unitary matrices here are in fact real and orthogonal matrices.

Intrinsic bond orbitals were introduced by Knizia^2^ and are the result of maximizing the PM functional,

3 $$L^{\left(\mathrm{PM}\right)}\left[\left\{u_{ij}\right\}\right]=\overset{N_{\mathrm{occ}}}{\underset{i}{\sum }}\overset{N_{\mathrm{atoms}}}{\underset{A}{\sum }}\left|\left\langle W_{i}\right|P_{A}\right|W_{i}\rangle {|}^{2}$$

where **P**_A_ = ∑_μϵA_|μ⟩⟨μ| are projectors onto a certain set of atom-centered functions |μ⟩, also known as intrinsic atomic orbitals (IAOs). This choice provides an unbiased measure of atomic partial charges and addresses the well-known basis set dependence associated with Mulliken populations. While alternative partial charge estimates have been proposed to address this issue for molecules^31^ and also for periodic systems,^20^ IAO-based charges also independently demonstrated their ability to accurately characterize bonding even in nontrivial transition structures of chemical reactions.^3^ In passing we note the often-overlooked near-equivalence of quasiatomic minimal-basis-set orbitals (QUAMBOs)^32^ and IAOs.^33^ Figure 1 illustrates examples of IBOs for a selection of materials. These visualizations are qualitatively consistent with previously reported generalized Wannier orbitals derived from Pipek-Mezey type localization functionals.^20–23^

**Figure 1:**
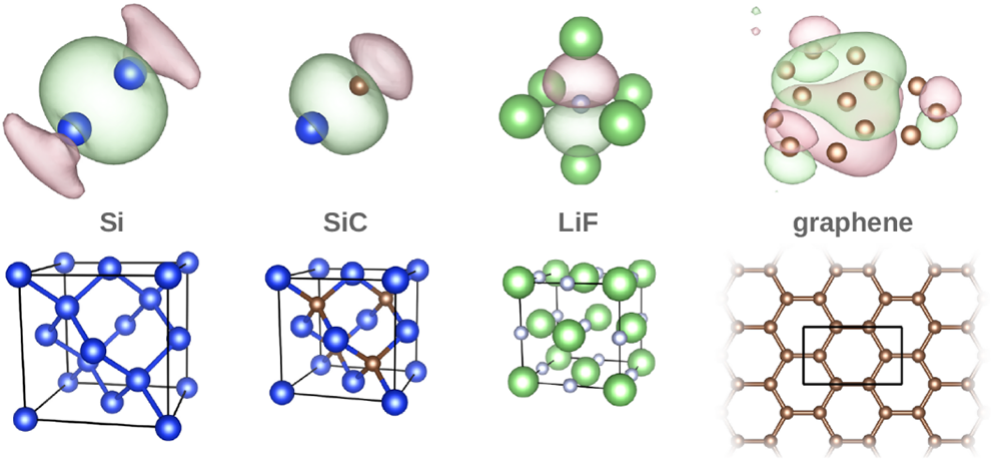
Visual representation of intrinsic bond orbitals (IBOs) in a selection of materials. The top row shows an IBO only with those sites of the periodic structure it connects, indicating the bond. The bottom row shows the conventional unit cell of the corresponding material. All pictures were made with VESTA,^26^ using an isosurface level of 5.0 for the orbitals.

The IAOs can be constructed from any set of atomic functions |*f*_μ_⟩ via the projection:

4 $$\left|\mu^{\mathrm{IAO}}\right\rangle =\left(1+O-\overset{∼}{O}\right)\left|f_{\mu }\right\rangle$$

where O is the projector onto the occupied space and $\overset{∼}{O}$ projects onto the space spanned by occupied orbitals from a minimal atomic basis. These projectors are defined as

5 $$O=\overset{N_{\mathrm{occ}}}{\underset{i}{\sum }}\left|\chi_{i}\right\rangle \left\langle \chi_{i}\right|,\;\overset{∼}{O}=\overset{N_{\mathrm{occ}}}{\underset{i}{\sum }}\left|{\overset{∼}{\chi }}_{i}\right\rangle \left\langle {\overset{∼}{\chi }}_{i}\right|$$

with the orbitals $\left|{\overset{∼}{\chi }}_{i}\right\rangle$ given by

6 $$\left|{\overset{∼}{\chi }}_{i}\right\rangle =\mathrm{orth}\left[\underset{\mu \nu }{\sum }\left|f_{\mu }\right\rangle S_{\mu \nu }^{-1}\langle f_{\nu }\left|\chi_{i}\right\rangle \right]$$

where *S*_μν_ = ⟨ ***f***_μ_| ***f***_ν_⟩ is the overlap matrix of the atomic functions and ″orth″ denotes orthogonalization. For the atomic functions |*f*_μ_⟩ we use (DFT) orbitals of the free atoms.^9^ While our definition of Intrinsic Atomic Orbitals (IAOs) in eq 4 differs from Knizia’s original formulation, they are equivalent when the minimal atomic basis is a subspace of the main basis. This condition is satisfied for a plane wave basis as a main basis, as the minimal atomic basis is also represented within it. The minimal atomic basis orbitals $\left|{\overset{∼}{\chi }}_{i}\right\rangle$ approximate the occupied orbitals, while the exact occupied mean-field orbitals $\left|\chi_{i}\right\rangle$ are obtained from a preceding mean-field calculation in the plane wave basis. The term $O-\overset{∼}{O}$ in eq ([Disp-formula eq4]) augments the atomic functions to form the IAOs, ensuring completeness of the occupied space. As long as no occupied orbital is orthogonal to the atomic functions, i.e., $\underset{\nu }{\sum }\left\langle f_{\nu }\right|\chi_{i}\rangle \neq 0$ , ∀*i*, the IAOs form an exact atom-centered basis for the occupied space.

### 2.2 Riemannian Construction of Intrinsic Bond Orbitals

Efficient optimization algorithms are vital for the success of localization methods, relying on the optimization of an orbital-dependent cost function L . As described previously in Sec. 2, we employ the IBO method. Optimization is performed using a Riemannian geometry approach, exploiting the topological properties of the unitary group to preserve the unitary constraint inherently. The search directions are translated to geodesics on the manifold, leading to more efficient optimization steps. Early works on these topics were conducted, for example, by Luenberger and Gabay.^34,35^

#### 2.2.1 Riemannian Optimization Under Unitary Constraint

The use of Riemannian optimization for problems with unitary constraints is an established field, and the theory described in this section is not novel. It serves to summarize the essential mathematical background required to understand and implement the algorithms used in this work. Our discussion largely follows the foundational literature on the topic, including the key works of Abrudan et al.^36,37^ Huang et al.,^38,39^ and Edelman et al.^40^

We opted for a Riemannian optimization approach due to its inherent suitability for handling unitary matrix constraints. Unlike traditional Euclidean methods that struggle to maintain unitarity and often suffer from slow convergence, Riemannian optimization operates directly on the manifold of unitary matrices. An illustrative comparison of how Riemannian and Euclidean algorithms operate under the unitary constraint were provided by Abrudan et al.^36^ The Riemannian approach respects the inherent “curved space” nature of the parameter space, allowing optimization along geodesics—the most efficient paths on this manifold. Furthermore, by recognizing that unitary matrices form a Lie group under multiplication, we leverage the algebraic properties of this group to ensure unitarity is preserved throughout the optimization process. This avoids the need for costly restoration steps or penalty functions, leading to more accurate and efficient convergence.

#### 2.2.2 Unconstrained Optimization

In this section, we introduce the concept of unconstrained line search algorithms, which are later adapted for application on manifolds. A minimum (or maximum) of some function L(U) is approached iteratively, where ***U*** represents an abstract vector in the parameter space. The optimization algorithms we compare are Conjugate-Gradient (CG), limited-memory BFGS­(L-BFGS) and Steepest Ascent (SA) solvers, in this paper we focus particularly on the first two, as SA has proven to be clearly inferior in our calculations and in refs. 21,23 Line search algorithms select a suitable direction in parameter space in a first step and subsequently determine an optimal step size along that chosen path. A detailed treatment of these topics can be found in ref 41 Without constraints, these algorithms are unrestricted within the respective parameter space, and follow the general update formula

7 $$U_{k+1}=U_{k}+\alpha_{k}H_{k}$$

where the iterates ***U***_k+1_ and ***U***_*k*_ are estimates of the desired extremum, α_*k*_ is the step size ***H***_*k*_ is the search direction.

For SA, the search direction ***H*** is chosen as the gradient $\nabla L\left(U_{k}\right)$ . The CG search direction is calculated according to the formula:

8 $$H_{k}=\nabla L\left(U_{k}\right)+\beta_{k}H_{k-1}$$

where β is a weighting factor that uses information from the previous step. Based on the work from Lehtola et al.^19^ we use the Polak-Ribíre (PR) formula for the factor β_*k*_.^42,43^ The initial search direction is $H_{0}=\nabla L\left(U_{0}\right)$ . BFGS^44–47^ is a quasi-Newton algorithm that mimics Newton’s method of minimizing the second-order Taylor series of the cost function. The Newton search direction is $H_{k}=-B_{k}\nabla L\left(U_{k}\right)$ with the inverse Hessian ***B***_*k*_. For high-dimensional problems, the computational cost of calculating the Hessian or its inverse is usually prohibitively high, so quasi-Newton algorithms aim for an accurate approximation. The L-BFGS approximation is given by

9 $$B_{k+1}=\left(I-\rho_{k}y_{k}s_{k}^{T}\right)B_{k}\left(I-\rho_{k}y_{k}s_{k}^{T}\right)+\rho_{k}s_{k}s_{k}^{T}$$

where ***I*** is the identity,

10 $$\rho_{k}=\frac{1}{y_{k}^{T}s_{k}}$$

and

11 $$s_{k}=U_{k+1}-U_{k},\;y_{k}=\nabla L_{k+1}-\nabla L_{k}$$

A requirement for the existence of a solution is the so-called curvature condition $s_{k}^{T}y_{k}>0$ , which ensures that the Hessian is positive definite and therefore invertible. This can be ensured by using a step size algorithm that is based on the Wolfe conditions^41^ for example. Note that the vectors in eqs 9 to 11 are not necessarily one-dimensional objects. As discussed later, in our use case we treat unitary matrices as abstract vectors with the corresponding Frobenius product serving as inner product.

L-BFGS^48^ is an approximation of BFGS, designed specifically for high-dimensional problems. While BFGS stores ***B*** explicitly, the limited memory version L-BFGS^48^ approximates eq 9 iteratively, therefore only retaining vectors ***y*** and ***s*** from a fixed number of previous iterations (memory). In our case, ***y*** and ***s*** are matrices of size *n* × *n*, making ***B*** of size *n* ^4^. This large scaling restricts BFGS to small systems, while L-BFGS is usually the method of choice for large-scale problems with a high-dimensional parameter space. With increasing memory size, the L-BFGS approximation approaches BFGS and if every step is stored they are mathematically equivalent.

When setting the memory size to 1, L-BFGS is closely related to CG methods, which also memorize the gradient at the previous point to update the search direction.^41^

After finding a search direction applying one of the above methods, a suitable step size has to be selected to determine an exact point along that path. Step size algorithms are in general independent of the way search directions are selected, although some are more suitable than others. Abrudan et al. suggest interpolating along the search path and calculating the maximum (minimum) of the resulting polynomial^37^ to obtain a reasonable estimate.

#### 2.2.3 Riemannian Geometry

A very elegant way to impose constraints on parameters is to exploit topological properties of the parameters. For a more detailed treatment of the concepts in this chapter, especially in the context of optimization, consider refs 37, 39, and 49.

We introduce Riemannian manifolds, smooth manifolds equipped with a metric. In general, a smooth manifold M is a topological space that fulfills special requirements regarding distance, neighborhood and differentiability. To each point U∈M , a tangent space $T_{U}M$ is attached, i.e., the set of all possible tangent vectors at that point.

Consider a smooth curve

12 γ(t):R→M,⁣γ(0)=U

If M is a submanifold of Euclidean space, a tangent vector ***X*** to M at point ***U*** is intuitively defined as the derivative of this curve at *t* = 0,

13 $$X_{U}\equiv \frac{d}{dt}\gamma \left(t\right){|}_{t=0}$$

In this sense, a tangent vector defines the direction of a curve on the manifold.

A textbook example for a mapping procedure between tangent spaces and the manifold itself is the exponential map (see eq 16), which enables movement along curves.

Riemannian manifolds are equipped with a Riemannian metric, defined on each tangent space as inner product _g_(***X***, ***Y***) =⟨ ***X***, ***Y*** ⟩, where ***X***, ***Y*** are tangent vectors.

#### 2.2.4 The Unitary Group *U*(*n*)

A key property of unitary *n* × *n* matrices is that they form a Lie group *U*(*n*), with matrix multiplication as a group action. The tangent space of the point at unity is highlighted as the Lie algebra of the group, $T_{I}U\left(n\right)\equiv u\left(n\right)$ , consisting of all skew-hermitian *n* × *n* matrices.

The group action defines two maps, known as *right translation* and *left translation*, meaning the multiplication of a point ***V*** ϵ *U*(*n*) by another point on the right:

RU:U(n)→U(n)V→VU≡V′ 14

and equivalently for left translation.

A tangent vector *X_**V**_* ϵ *T_**V**_U*(*n*) can be translated in the same way to another tangent space *T_**V**__U_U*(*n*):

RU*:TVU(n)→TV′U(n)XV→XVU≡XV′ 15

Importantly, these translations are isometries with respect to the Riemannian metric, so distances are preserved, allowing for the simple movement of curves and tangent vectors between points on the manifold. Following eq 15, every vector in the Lie algebra $X_{I}\in u$ can be moved to any tangent space *T_U_U*(*n*) by multiplication with ***U*** from the right, ***X** **_U_*** = ***X** **_I_** **U***, and vice versa every tangent vector can be easily translated to the Lie algebra: ***X** **_I_*** = ***X** **_U_** **U*** ^†^. This makes the Lie algebra a very convenient choice for calculations involving multiple tangent vectors.

The exponential mapping exp⁡:u→U(n) maps an element X∈u to the group, given by the matrix exponential

16 exp(αX)=γ(α)

where the curve γ:R→U(n) is a parametrized geodesic, the shortest path between two points of the group. This can be understood as taking a direction ***X*** and moving along the corresponding geodesic curve.

The concepts in this chapter are also valid for the orthogonal group *O*(*n*), consisting of orthogonal matrices as elements, while skew-symmetric matrices form the Lie algebra.

#### 2.2.5 Optimization on the Unitary Group

Combining the previously discussed ideas, optimization algorithms originally designed as unconstrained in the Euclidean parameter space can be generalized to Riemannian manifolds. Equipped with the Frobenius inner product as Riemannian metric, g­(***X***, ***Y***) =*Tr*(***X*** ^†^ ***Y***), the unitary group *U*(*n*) forms a Riemannian manifold.

According to Abrudan et al.^36^ the gradient of a function L:U(n)→R at some point ***U*** ϵ **U**(*n*) is given by

17 $$\nabla L\left(U\right)=\Gamma -U\Gamma^{†}U$$

where $\Gamma ≡dL/du_{ij}$ . Subsequently, this gradient is translated to the Lie algebra via right translation:

18 $$G\left(U\right)≡\nabla L\left(U\right)U^{†}=\Gamma U^{†}-U\Gamma^{†}$$

The algorithms SA, CG and BFGS are now introduced following section 2.2.2, utilizing the translated gradient ***G*** (***U***) to obtain the search direction ***H***_*k*_. This vector is mapped to the group using the exponential map in eq 16, the emanating curve exp­(α ***H**_k_*) is transported to ***U***_*k*_ to obtain ***U***_k+1_:

19 $$U_{k+1}=\exp\left(\alpha H\right)U_{k}$$

where the scaling factor α serves as step size.

In the case of L-BFGS, the fact that *U*(*n*) is a Lie group is especially advantageous. Vectors *y*_*i*_ and *s*_*i*_ do not need to be transported to the new iterate to calculate the search direction, as calculations can be performed in the Lie algebra.

## 3 Computational Methods and Implementation

The CG and SA solvers are implemented in the publicly available Julia package Lucon.jl (Loss optimization under unitary constraint)^30^ and in VASP as reported in ref..^9^ All considerations with the CG solver are performed using the Polak-Ribière update factor.^42^ The implementation of the L-BFGS solver was included in a development version of VASP in the scope of this work. Pseudocode for this algorithm is shown in Alg. 1, following the work of Huang et al. and Nocedal et al..^38,41^ The two-loop recursion was developed by Nocedal et al.^41^ and efficiently computes the L-BFGS search direction. For step size calculations, we utilize the method developed by Abrudan et al..^37^ To validate our implementation and confirm our results, we repeated all calculations using the manopt.jl package by Bergmann et al.^50,51^ where we selected a step size algorithm based on the Hager-Zhang scheme.^52,53^ The Euclidean derivative for the IBOs reads

20 $$\Gamma_{ij}^{\left(\mathrm{PM}\right)}=\frac{\partial L^{\left(\mathrm{PM}\right)}}{\partial u_{ij}^{*}}=2\overset{N_{\mathrm{atoms}}}{\underset{A}{\sum }}\overset{N_{\mathrm{occ}}}{\underset{k}{\sum }}\left\langle \chi_{i}\right|P_{A}\left|\chi_{k}\right\rangle u_{kj}|\overset{N_{\mathrm{occ}}}{\underset{lm}{\sum }}\left\langle \chi_{l}\right|P_{A}|\chi_{m}\rangle u_{lj}^{*}u_{mj}|$$

The HF orbitals $\left|\chi_{i}\right\rangle$ are obtained from the plane-wave based Vienna Ab initio Simulation Package­(VASP)^27–29^ using the PAW method.^54^ The PAW pseudopotentials use a frozen core and are provided as POTCAR files with VASP, see Table 1. For each material, the largest ENMAX value was multiplied by a factor of 1.25, and then rounded up to the nearest multiple of ten to determine the plane wave cutoff ENCUT in units of eV. By scaling the default value (ENMAX) in this way, we ensure that we use a sufficiently large base for each material. For example, the calculations for SiC were performed using ENCUT=⌈max­(413.992,319.379)·1.25⌉_10_ =⌈517.490⌉_10_ =520, where ⌈x⌉_10_ denotes rounding *x* up to the nearest multiple of 10. The singularity of the Coulomb potential in reciprocal space is treated via the truncation method introduced by Spencer and Alavi in ref 55 Supercells are considered using a **Γ**-only sampling of the BZ. The atomic structures for caffeine, benzene, coronene, graphene with flower defect, and silicon with interstitial defect can be found in the Supporting Information.

**Table 1:** Used POTCAR Files, Defining the PAW Pseudopotential as well as the Plane Wave Cutoff^a^

Element	POTCAR header	valence	ENMAX (eV)
H	PAW_PBE H_GW 21Apr2008	1s^1^	300.000
Li	PAW_PBE Li_AE_GW 25Mar2010	1s^2^2p^1^	433.699
B	PAW_PBE B_GW_new 26Mar2016	2s^2^2p^1^	318.614
C	PAW_PBE C_GW_new 19Mar2012	2s^2^2p^2^	413.992
N	PAW_PBE N_GW_new 19Mar2012	2s^2^2p^3^	452.633
O	PAW_PBE O_GW_new 19Mar2012	2s^2^2p^4^	434.431
F	PAW_PBE F_GW_new 19Mar2012	2s^2^2p^5^	480.281
Na	PAW_PBE Na_sv_GW 11May2015	2s^2^2p^6^3p^1^	372.853
Mg	PAW_PBE Mg_GW 13Apr2007	3s^2^	126.143
Al	PAW_PBE Al_GW 19Mar2012	3s^2^3p^1^	240.300
Si	PAW_PBE Si_GW_nc 03Jul2013	3s^2^3p^2^	319.379
P	PAW_PBE P_GW 19Mar2012	3s^2^3p^3^	255.040
Cl	PAW_PBE Cl_GW 19Mar2012	3s^2^3p^5^	262.472
Ti	PAW_PBE Ti_sv_GW 05Dec2013	3s^2^3p^6^3d^4^	383.774
Ga	PAW_PBE Ga_GW 22Mar2012	4s^2^4p^1^	134.678
Ge	PAW_PBE Ge_GW 04Okt2005	4s^2^4p^2^	173.807
As	PAW_PBE As_GW 20Mar2012	4s^2^4p^3^	208.702

a For every material, the largest ENMAX value was scaled by a factor of 1.25 to define the plane wave cutoff ENCUT.

We also implemented the DIIS technique to investigate its potential for accelerating convergence to the optimum. The DIIS technique is a mixer that seeks to find optimal linear combinations of previous iteration steps. This technique is well-established for accelerating iterative solvers in finding the HF ground state. When finding an optimal unitary matrix, this matrix must be parametrized to construct linear combinations of previous solutions, resulting in a new unitary matrix. While several parametrizations exist^56^ we used the exponential parametrization, which was already successfully applied for the rotation of orbitals in previous works.^57^ In the case of unitary (orthogonal) rotations, we write *U* =e^iΘ^ (*U* =e^Θ^) with the hermitian (skew-symmetric) matrix Θ containing the rotation parameters. Note that this consideration no longer follows the idea of a Riemannian optimization, but is necessary to mix parameters (here Θ) in the DIIS mixer. Our implementation was modeled after the documentation by C. D. Sherrill.^58^ Accordingly, we define the error vectors of the DIIS scheme as Δ*_i_* = Θ*_i_* – Θ*_i_*_–1_ and find the optimal parameters $\Theta_{\mathrm{opt}}=\overset{n}{\underset{i=1}{\sum }}\tau_{i}\Theta_{i}$ by minimizing the Frobenius norm of $\Delta =\overset{n}{\underset{i=1}{\sum }}\tau_{i}\Delta_{i}$ , where *n* represents a fixed history size. The optimal parameters Θ_opt_ themselves are never added to the history to avoid linear dependencies.
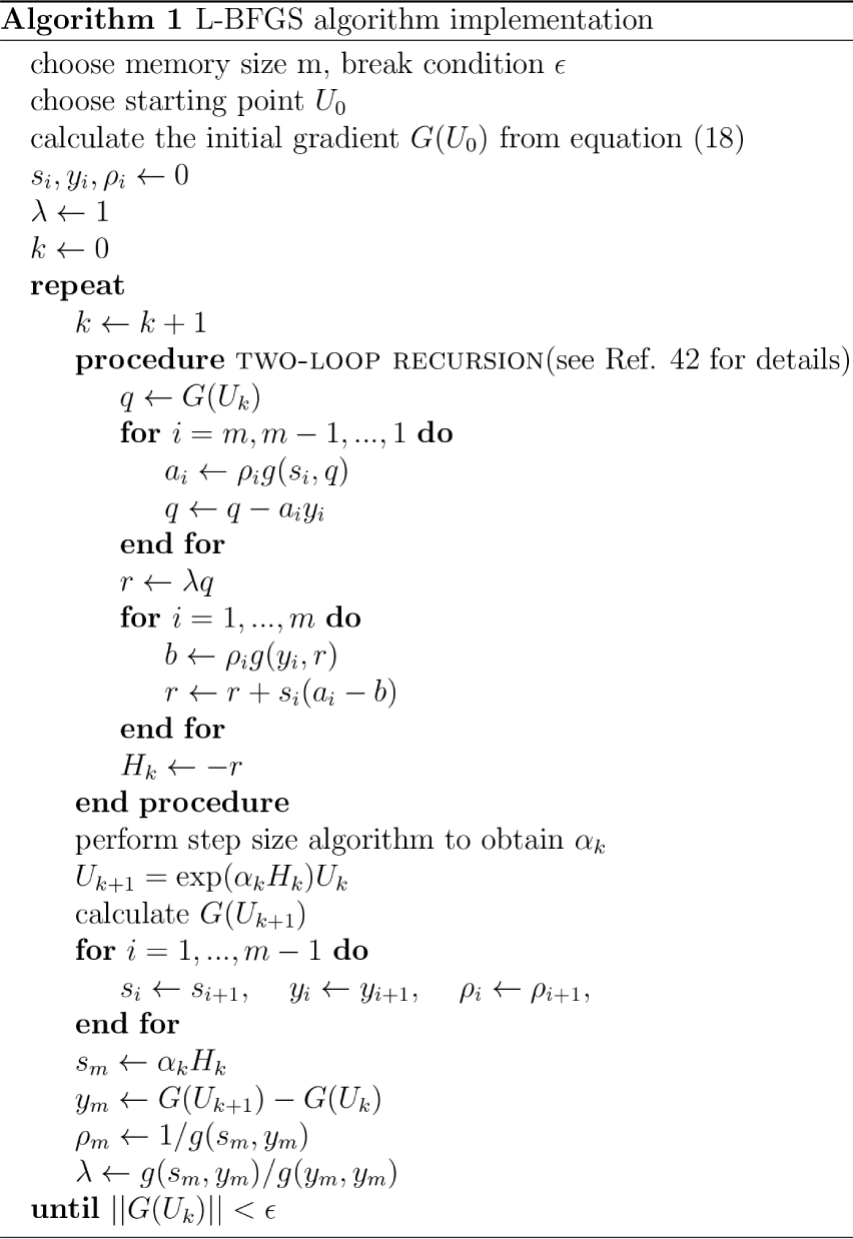


## 4 Results

We performed computations for several molecules, molecular crystals, bulk solids and systems with broken translational symmetry. A special focus was on large supercells. If not stated otherwise, the calculations were initialized with random unitary matrices and a break condition for the gradient norm of $\left|\right|G\left|\right|=\sqrt{\left\langle G,G\right\rangle }<10^{-5}$ was chosen as the convergence criterion. We measure the *performance* of an algorithm by the number of iterations required to reach convergence.

Surprisingly, specifically for periodic systems, large L-BFGS memory sizes do not necessarily lead to improved performance for IBO localization, as shown in Figure 2 for a graphene flower defect system (supercell with 324 occupied orbitals). However, the statistical variance of the required number of iterations decreases with higher memory, while the increase in computational cost is negligible. If not stated otherwise, a fixed memory size of 20 is used for our L-BFGS calculations. Note that the performance of CG and L-BFGS is similar for this example, an observation that is consistent across all periodic systems tested.

**Figure 2:**
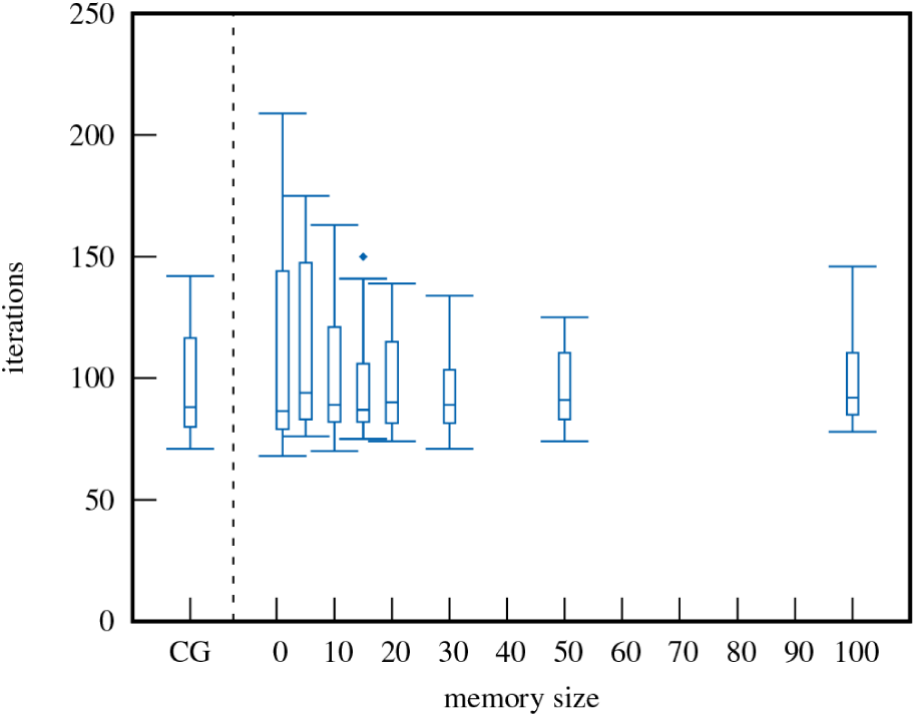
Box-and-whisker plot of the number of required iterations against the number of memorized L-BFGS steps compared to CG for a graphene supercell (162 atoms) with flower defects.

Figure 3 shows the median number of necessary iterations against the system size for a selected set of systems using the L-BFGS solver. The scaling of the iterations with system size is roughly proportional to the fourth root of the number of occupied orbitals *n*, i.e., sublinear, illustrated by the dashed lines.

**Figure 3:**
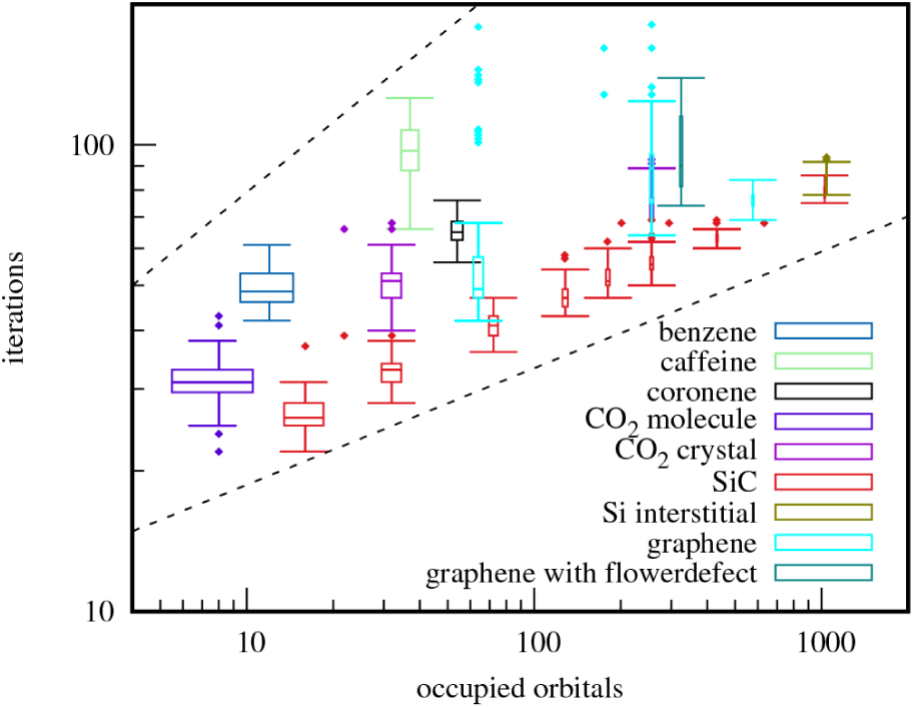
Box-and-whisker plot of the L-BFGS iterations against occupied orbitals *n* for several systems and supercell sizes. The dashed lines are proportional to $\sqrt{n}$ and $\sqrt[{4}]{n}$ respectively, giving an idea of scaling.

In Table 2, the median number of required iterations are listed for L-BFGS, CG and SA algorithms. Interestingly, for supercells with broken symmetry, L-BFGS and CG show a performance similar to that for the pristine case, as the results indicate for the flower defect graphene and Si with interstitial defects. L-BFGS and CG outperform SA for all test systems, L-BFGS has an advantage over CG only for molecules.

**Table 2:** Median Number of Required Iterations for L-BFGS , CG, and SA Solvers for Several Systems of Various Cell Size (Number of Occupied Orbitals)

material/molecule	#occ	L-BFGS	CG	SA
benzene	12	49	83	7093
caffeine	37	97	132	3217
coronene	54	65	85	671
CO_2_ molecule	8	31	38	171
CO_2_ crystal	32	51	53	269
	256	73	81	316
SiC	16	26	26	54
	32	33	33	68
	72	41	41	87
	128	47	45	102
	180	51	50	110
	256	56	53	120
	432	63	58	134
defect Si	1040	84	74	154
graphene	64	50	51	124
	256	89	78	197
	576	76	67	155
flower defect graphene	324	90	88	265

Notably, some graphene cells exhibit outliers with a substantially higher number of iterations, approaching other local extrema. This behavior, visible in Figure 3, is further quantified in Table 3 for the L-BFGS algorithm. For example, flower defect graphene calculations (324 occupied orbitals) converge to a slightly worse maximum in 38% of runs. Similar observations were made with the manopt.jl package considering CG and L-BFGS, using a different line search method.

**Table 3:** For , the Algorithms Not Always Converge to the Same Value of the Cost Function^a^

		largest maximum		second largest maximum	
material	#occ	iterations	cost per #occ	iterations	cost per #occ
graphene	64	49 (92%)	1.1357	112 (8%)	1.1278
	256	87 (90%)	1.1346	126 (7%)	1.1293
	576	76 (100%)	1.1430	0 (0%)	
flower defect	324	83 (62%)	1.1350	119 (38%)	1.1341

a The percentage of runs (out of 60 each) converging to the respective maximum is given in brackets. Notably, when converging to a lower value, the number of iterations is considerably higher. These outliers are also visible in Figure 3 for the graphene cells.

As listed in Table 4, metal oxides require about 1 order of magnitude more iterations for a fixed convergence threshold than other systems. Here, all considered localization functionals IBO, FB and VN show a similar trend. The convergence behavior of the L-BFGS optimization is illustrated in Figure 4, which displays the gradient norm per iteration for a TiO_2_ supercell containing 72 atoms and 288 occupied orbitals. An initial rapid reduction in the gradient norm is observed within the first 50–100 iterations, transitioning to a slower, more irregular decrease accompanied by significant oscillations.

**Table 4:** Comparison of the Average Number of Necessary Iterations for Metal Oxides and a Set of Other Materials Employing the intrinsic bond orbital (IBO), Foster-Boys (FB), and von-Niessen (vn) Localization Functionals^a^

material	oxide	#occ	IBO	FB	VN
SiC	nonoxide	256	53	140	39
CO_2_	molecular oxide crystal	256	81	546	294
SiO_2_ (α-quartz)	nonmetal oxide	288	155	496	154
TiO_2_ (rutile)	metal oxide	288	678	1628	605
MgO	metal oxide	288	1737	1464	365

a Supercells containing a comparable number of occupied orbitals were considered. The average was calculated from 4 runs initialized with random unitary matrices.

**Figure 4:**
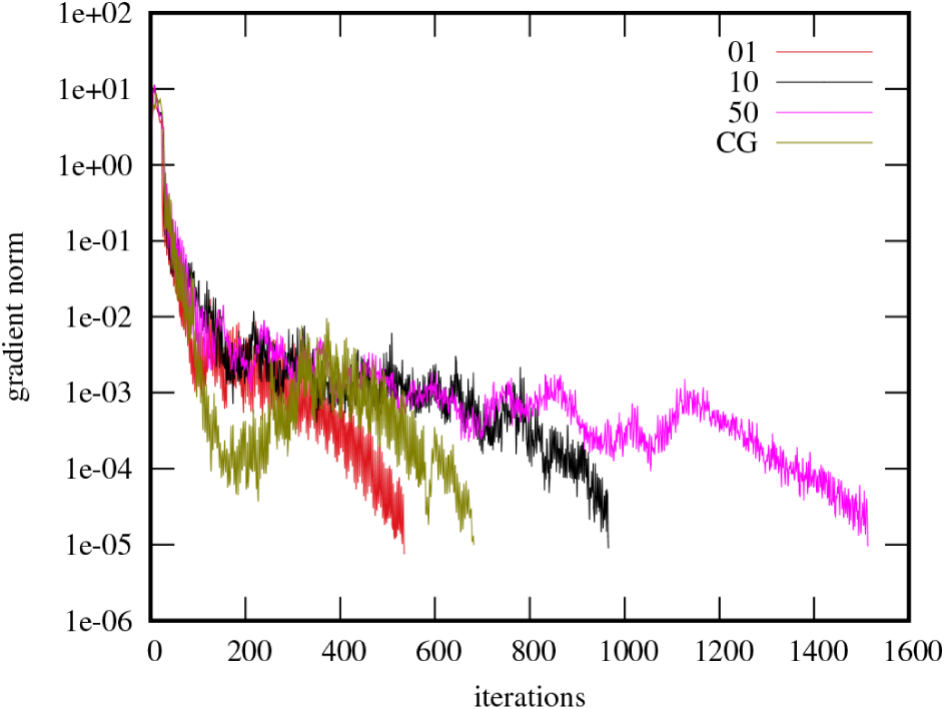
Convergence of L-BFGS with several memory size settings and CG, for a TiO_2_ supercell (72 atoms, 288 occupied orbitals), the unity matrix serves as starting point (i.e., starting from Bloch orbitals).

In contrast, a typical convergence pattern of nonoxides is shown in Figure 5 using the aforementioned graphene flower defect supercell as an example. From a certain iteration step onward (in this case somewhere between 50 and 60 iterations), convergence is consistently exponential.

**Figure 5:**
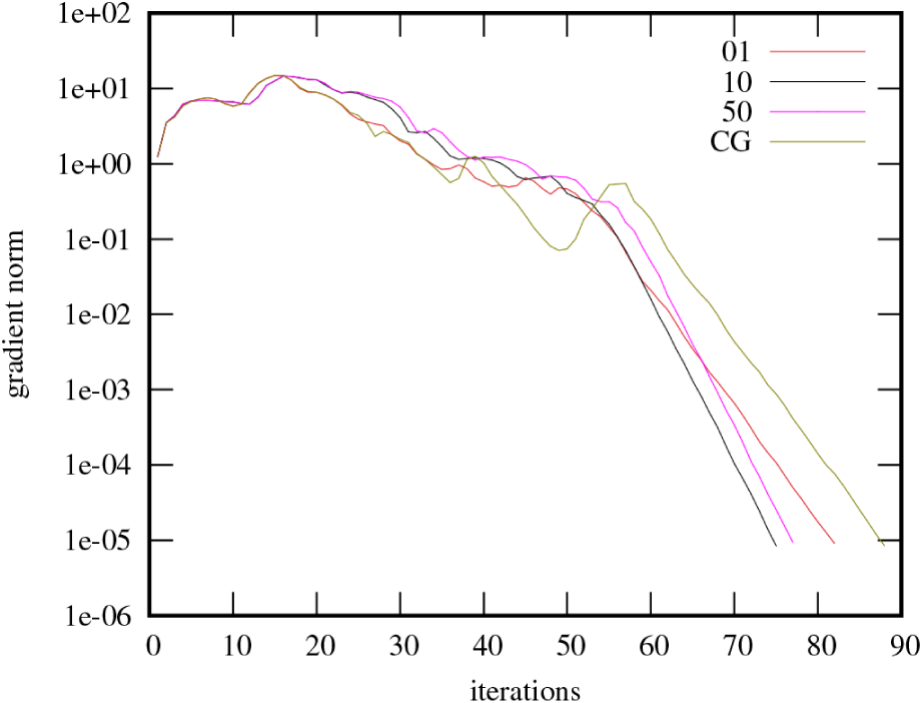
Convergence of L-BFGS with several memory size settings and CG, for a graphene supercell (162 atoms) with flower defects, the unity matrix serves as starting point (i.e., starting from Bloch orbitals).

The largely cubic scaling of the L-BFGS runtime per iteration and the system size is depicted in Figure 6 for SiC. Except for very small cells, where the impact of several inexpensive routines is visible, the runtime scales proportionally to *n* ^3^, where *n* is the number of occupied orbitals. This behavior is expected, as *n* determines the size of most of the involved matrices and consequently the cost of matrix operations. CG and SA are only marginally faster, on average by 3.5% and 4.1%, respectively, disregarding the two smallest cells. It can be stated that the additional complexity of L-BFGS is insignificant in relation to the cost of other routines like gradient calculation or line search algorithm.

**Figure 6:**
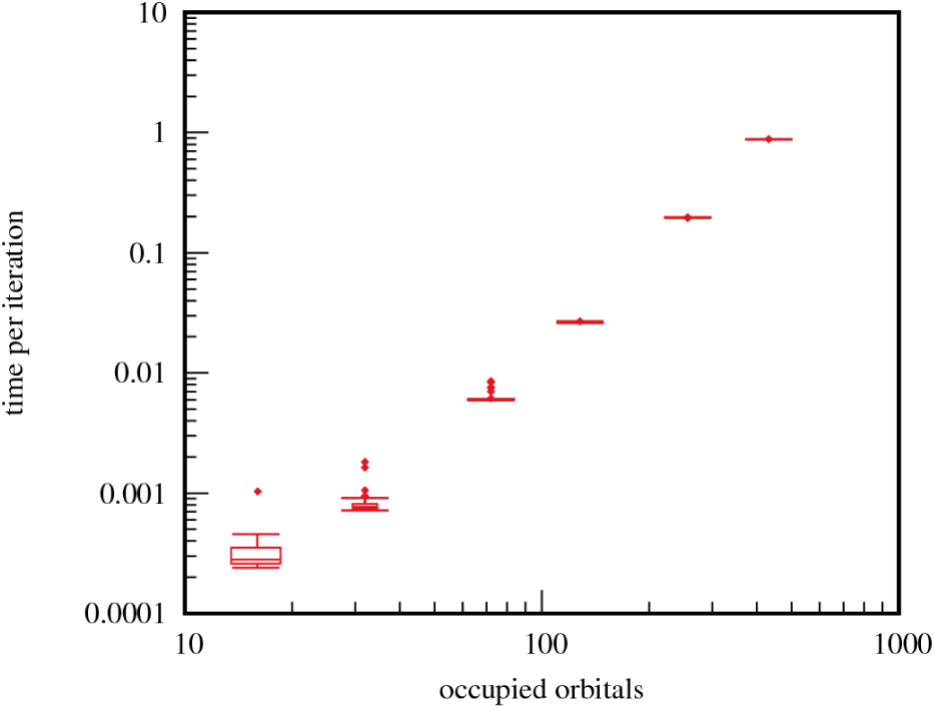
L-BFGS runtime per iteration in seconds against occupied orbitals, *n*, of SiC supercells, showing a *n* ^3^ scaling.

In order to assess whether the DIIS technique can accelerate the convergence of our Riemannian solvers, we considered a supercell of SiC containing 256 occupied orbitals. We start the DIIS mixer when the gradient norm fell below a certain threshold using a fixed history size of 10. As evident in Figure 7 the DIIS technique can beat the convergence of the SA algorithm using a threshold of 10^–1^. A clear distinction is visible between an already filled history (thick lines) and the case when the mixer starts from an empty history (thin lines). Increasing the threshold to 10 ° leads to a slightly worse convergence behavior. When the DIIS technique is activated following a certain number of iterations (here *i* ≥40), it exhibits poor convergence behavior from a suboptimal initial state, necessitating 719 iterations to achieve convergence. Furthermore, the DIIS mixer is unable to accelerate the convergence of the CG solver in combination with the Polak-Ribiére (PR) update factor. This also applies, unfortunately, to the challenging case of metal oxides. We note that the DIIS solution is always updated by the SA solver, i.e., the label “CG+DIIS” in Figure 7 denotes a CG solution until the threshold is reached, followed by the SA solver with DIIS mixing. This is due to the fact that updating the DIIS solution using CG with PR factors consistently leads to a nonconverging behavior.

**Figure 7:**
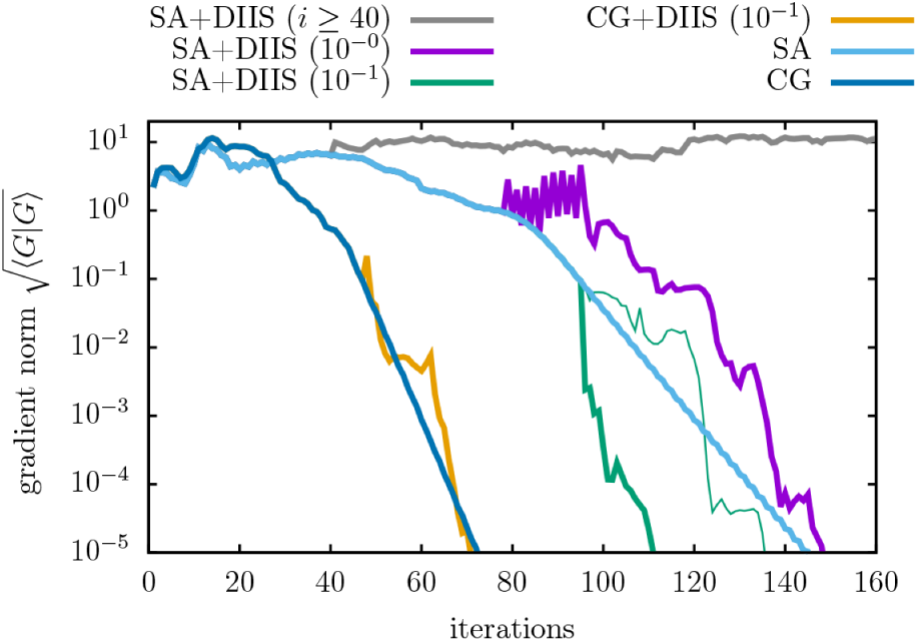
Convergence of the DIIS solver (memory size 10) for a SiC supercell with 256 occupied orbitals. The gradient norm or iteration threshold for initiating DIIS is shown in brackets. Thin lines represent the case when the DIIS starts with an empty history. All calculations use the identity as the starting point, i.e., Bloch orbitals.

## 5 Discussion

5In this first part of the paper, we consider the performance of various solvers, avoiding any bias such as initial guesses. We observed that both L-BFGS and CG exhibited similar performance and significantly outperformed the SA solver within their respective Riemannian formulations. The comparable performance of our CG and L-BFGS implementations, coupled with the latter’s limited sensitivity to memory size, suggests potential limitations in the L-BFGS Hessian approximation specifically within the context of IBO localization. This contrasts with FB localization, where larger L-BFGS memory sizes have been shown to improve performance and L-BFGS consistently outperforms CG.

L-BFGS iterations are only marginally slower than CG and SA in runtime measurements, dispelling a potential disadvantage and indicating the runtime dominance of other routines like gradient calculation or step size search. Runtime per iteration scales cubically with the number of occupied orbitals.

For graphene supercells, both pristine and defect-containing, we observed that multiple stochastically initialized runs converged to distinct, suboptimal local maxima of the cost function. This problem is consistent across all solvers tested and indicates a significant presence of local extrema and saddle points in the optimization landscape, a common challenge for high-dimensional cost functions.

We also observe that the localization procedure for the metal oxides MgO and TiO_2_ requires significantly more iterations to converge compared to other systems examined. While these materials exhibit strong ionic character, this characteristic alone does not explain the observed slow convergence. Specifically, we did not encounter similar convergence difficulties with other ionic systems such as LiF and NaCl, which share the same crystal structure as MgO. The primary distinguishing features of MgO and TiO_2_ compared to the other systems are the ionic nature in combination with the −2 charge of the anion and the metallic nature of the cation.

Despite its effectiveness in accelerating the convergence of the SA solver at sufficiently low gradient norms (below 10^–1^), the DIIS mixer did not yield a comparable improvement for the CG solver. Furthermore, the convergence difficulties encountered with metal oxides remained unaffected by the application of the DIIS mixer.

Our results also present a noteworthy divergence from those of Clement et al.^21^ as briefly noted in the Introduction. They report a significant performance advantage for the Riemannian L-BFGS over the CG solver for any of the materials considered in their work. This discrepancy is particularly pronounced in graphene, where Clement et al. reported approximately 3· 10^3^ iterations for CG convergence of PM orbitals, while our CG implementation convergences in less than 10^2^ iterations. Notably, our L-BFGS results align with those of Clement et al., showing comparable performance.

We speculate that the discrepancies for the CG solver could be due to the following different technical features of the implementations. For instance, while our approach uses large supercells sampled at the **Γ**-point only, Clement et al. employs small unit cells in combination with fine k-point sampling of the BZ. The **Γ**-point only approach allows us to use real-valued orbitals in the real-space basis, avoiding the gauge freedom with complex phases and the challenging search for a smooth gauge.^17^ Furthermore, while we restrict the optimization to valence electrons using a frozen core in combination with PAW pseudopotentials in the plane wave basis, Clement et al. follows an all-electron approach using Gaussian basis sets. A significant performance gain when restricting the optimization to valence electrons was already reported by Zhu et al.^23^ Further differences of the algorithms include the starting point and the partial charge estimate for the PM localization functional. Both works use a similar line search strategy, based on a polynomial approximation approach.^37^

## 6 Properties of Intrinsic Bond Orbitals

In the second main part of the paper, we investigate spatial properties of (IBOs) for a variety of insulating solids, as listed in Table 5. These IBOs were constructed from periodic Hartree–Fock (HF) orbitals, and their spatial character was analyzed in relation to the experimental lattice constant and band gap. A focus was the average orbital spread of the (IBOs) and its relationship with geometric properties, specifically the nearest neighbor distance (*d*_NN_) and the number of nearest neighbors (#NN). Our findings reveal that the orbital spread of the valence electrons per nearest neighbor distance, multiplied by the number of nearest neighbors, remains relatively stable across the materials considered. This can be captured by the empirical relation σ_HF_ ≈ 2.1α, where $\alpha =\frac{d_{\mathrm{NN}}}{\#\mathrm{NN}}$ . This trend was consistent across various crystal structures, independent of the band gap, providing a useful estimate for predicting the spatial extent of localized orbitals (see Figure 8).

**Table 5:** List of Considered Materials in Section 6.^a^

	structure	bond	a[Å]	d_NN_[Å]	#NN	σ_HF_[Å]	γ_exp_[*eV*]
C (Diamond)	A4	covalent	3.57	1.54	4	0.832	5.48
Si	A4	covalent	5.431	2.35	4	1.268	1.17
Ge	A4	covalent	5.652	2.45	4	1.351	0.74
NaCl	B1	ionic	5.569	2.78	6	0.813	9.50
MgO	B1	ionic	4.189	2.09	6	0.817	7.22
LiF	B1	ionic	3.972	1.99	6	0.676	14.5
SiC	B3	polar covalent	4.346	1.88	4	1.009	2.42
BN	B3	polar covalent	3.592	1.56	4	0.806	6.22
AlP	B3	polar covalent	5.451	2.36	4	1.204	2.51
GaAs	B3	polar covalent	5.64	2.44	4	1.307	1.52
GaN	B3	polar covalent	4.509	1.95	4	0.966	3.30
C (Lonsdaleite)	B4	covalent	4.347	1.54	4	0.835	3.35

a The structure column refers to the Strukturbericht Designation. The lattice constant a, the nearest neighbor distance dNN, the number of nearest neighbors #NN, the IBO orbital spread σHF, and the experimental band gap γexp are also provided.

**Figure 8:**
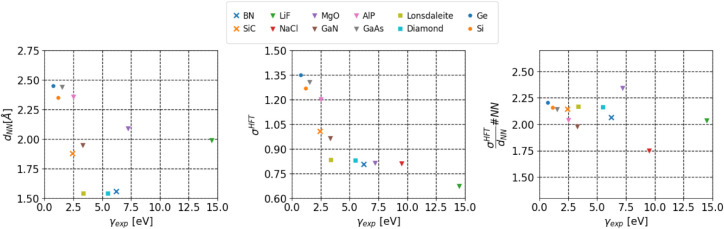
Plot of the nearest neighbor distance *d*_NN_ (left), the IBO orbital spread σ_HF_ (center), and relation between orbitals spread and $d_{\mathrm{NN}}/\#\mathrm{NN}$ (right) plotted against the experimental band gap of all considered materials.

We also analyzed the decay of the Fock exchange matrix entries,

21 $$K_{ij}=-\frac{1}{2}\int d^{3}r_{1}\int d^{3}r_{2}\frac{W_{i}^{*}\left(r_{1}\right)W_{j}\left(r_{1}\right)W_{j}^{*}\left(r_{2}\right)W_{i}\left(r_{2}\right)}{\left|r_{1}-r_{2}\right|}$$

in the basis of Wannier orbitals $W_{i}\left(r\right)$ in the form of IBOs. To this end, we define the error of the Fock exchange energy per atom as

22 $$\varepsilon =\left|\underset{ij}{\sum }K_{ij}-\overset{\mathrm{trunc}.}{\underset{ij}{\sum }}K_{ij}\right|/N_{A}$$

where *N*_*A*_ is the number of atoms. Two truncation methods were employed to estimate the error per atom, a magnitude cutoff and a distance cutoff. The magnitude cutoff eliminates matrix elements below a certain energy threshold (Figure 9), while the distance cutoff uses the interorbital distances of the centers of the IBOs to determine which elements to retain (Figure 10).

**Figure 9:**
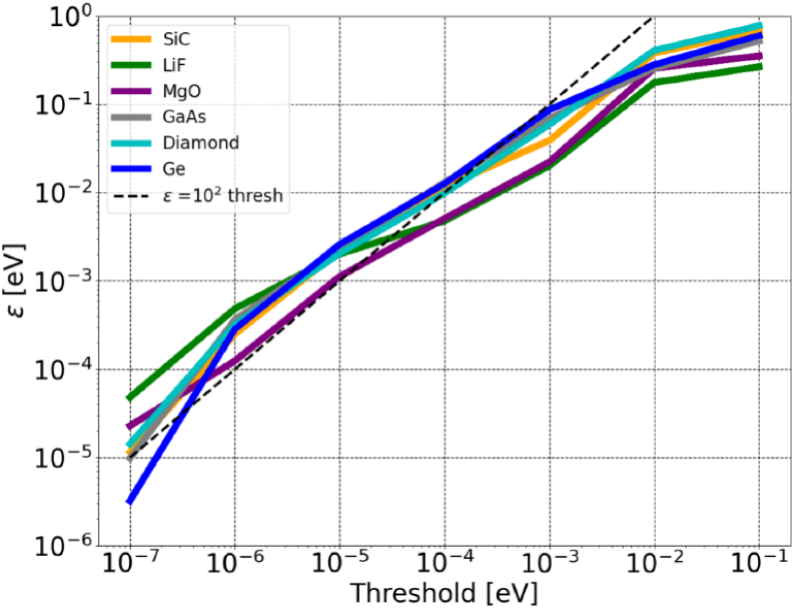
Error of the Fock exchange energy per atom *ε* for different thresholds using the magnitude cutoff.

**Figure 10:**
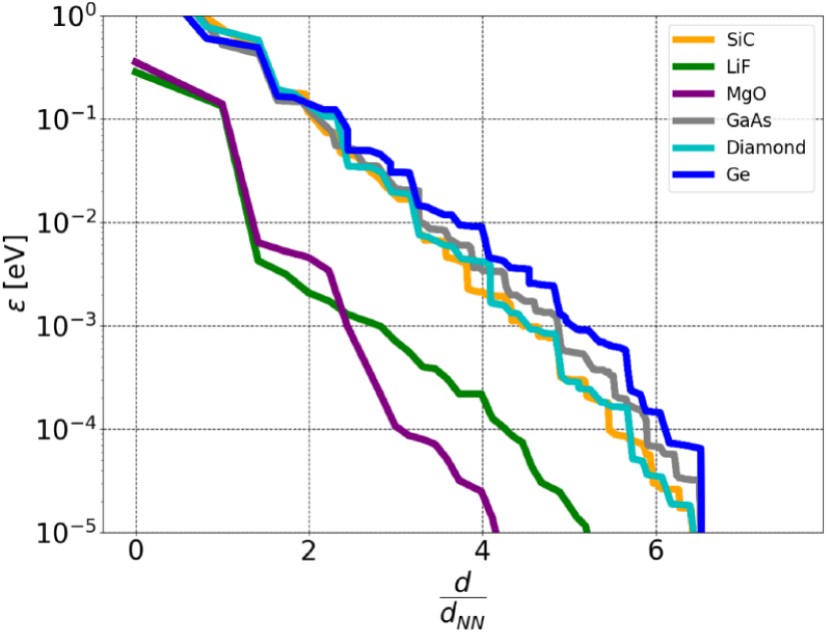
Error of the Fock exchange energy per atom with respect to the largest distance between the Wannier orbitals *d*_max_ per nearest neighbor distance, using the distance cutoff.

It is noteworthy that the magnitude cutoff method yields remarkably consistent error estimates across all considered materials. For example, comparing germanium, a narrow-gap semiconductor with an experimental band gap of 0.74 eV and high relative permittivity, to diamond, a wide-gap insulator with a band gap of 5.48 eV and significantly lower polarizability, reveals no substantial variation in error behavior. However, the distance cutoff method exhibits a distinct dependence for ionic compounds. In these materials, the valence charge is primarily localized on the anions, resulting in a depletion of valence electron density around the cations. Consequently, the interorbital distance between neighboring bonding orbitals increases to approximately 2d_NN_. This increased separation leads to a more rapid decay of the error when employing the distance cutoff for ionic systems.

Our findings on the truncation of the Fock exchange matrix further support the potential for computational savings due to its sparsity. This holds for both large-gap and small-gap materials, suggesting that the band gap has a relatively minor effect on the decay of matrix elements for practical purposes. Figure 11 shows the number of remaining nonzero Fock exchange matrix elements in dependence of *ε* for both methods.

**Figure 11:**
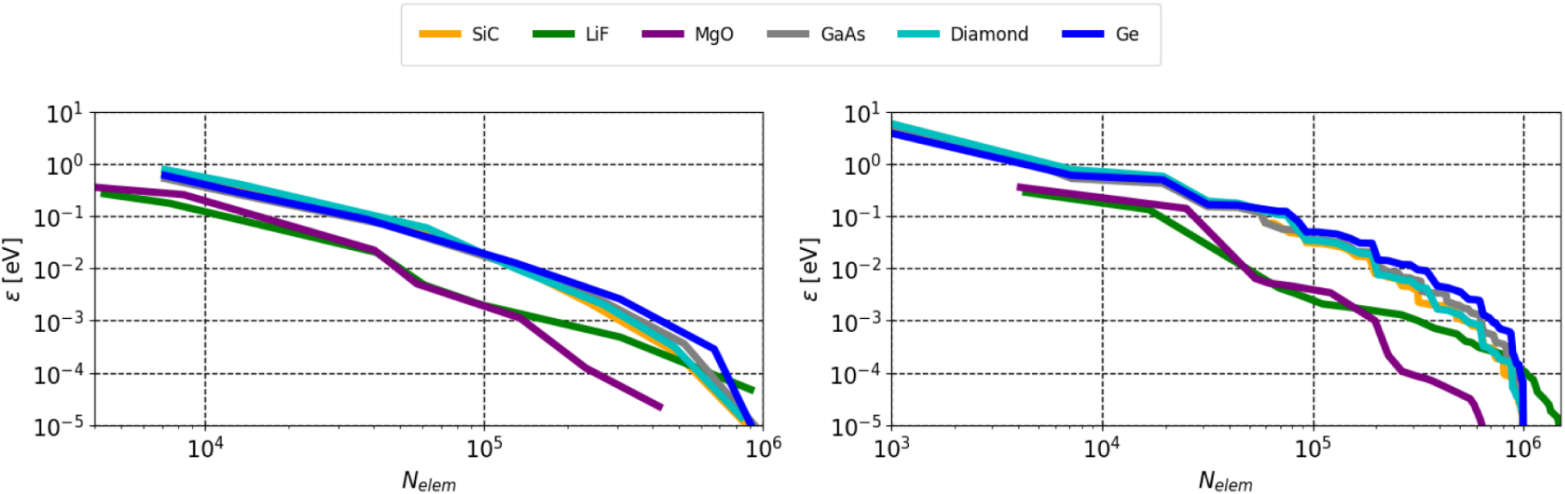
Fock exchange energy error per atom *ε* with respect to the number of nonzero elements *N*_elem_ of the Fock exchange matrix using the magnitude cutoff (left) and distance cutoff (right), as explained in the text. For our calculations the full Fock exchange matrix contains 1280^2^ ≈ 1.6 · 10^6^ matrix elements for LiF and 1024^2^ ≈ 10^6^ for all other materials in the plot. This corresponds to 4 X 4 X 4 supercells of the conventional cells.

In summary, the analysis of IBO properties across various bulk 3D materials, exhibiting covalent bonds, polar covalent bonds, and ionic bonds, reveals a correlation between orbital spread and geometric factors. This relationship provides a straightforward way to estimate the spatial extent of localized orbitals, which is crucial for the development of reduced-cost methods. Additionally, the truncation of the Fock exchange matrix demonstrates significant potential for reducing computational costs in large-scale simulations, with manageable errors across a wide range of materials.

## 7 Conclusion

In this work we studied the numerical construction and spatial properties of IBOs based on (HF) orbitals for a set of insulating solids. We reported a relation between the orbital spread measured in units of the nearest neighbor distance and the number of nearest neighbors. Independent of the band gap, this relation is relatively stable for all considered 3D semiconductors and insulators. It suggests that local methods based on the sparsity of Coulomb integrals can also be applied to materials with small band gaps without losing the sparsity. We verified this hypothesis for the particular case of the sparsity of the Fock exchange matrix in the basis of IBOs. Whether this can be extended to metallic solids or scenarios involving localized unoccupied orbitals, essential for many-electron correlation methods, remains an open question. This warrants further investigation in future work.

Additionally, we benchmarked various solvers to optimize the unitary matrix that transforms delocalized Bloch orbitals into localized Wannier orbitals, specifically in the form of IBOs. The solvers have been implemented within VASP and as a standalone open-source software package Lucon.jl.^30^ Our focus was on large simulation cells, which are crucial for realistic models, such as those involving surface phenomena and defects. Contrary to a previous study^21^ we did not observe a clear performance advantage of the L-BFGS solver, instead finding that both the CG and L-BFGS solvers exhibited similar performance. When using stochastic (unbiased) starting points, we found that constructing localized orbitals in supercells of metal oxides pose a significant challenge, requiring an order of magnitude more iteration steps than for the other materials considered. These findings underscore the importance of optimized initial guesses, the potential of effective preconditioning strategies, and the exploration of noniterative approaches for efficient Wannier orbital construction in solids.

## Supplementary Material



## Notes

Atomic structures used for the VASP calculations (PDF)
