# Supplementary material for: Convergence and Properties of Intrinsic Bond Orbitals in Solids
Source: J Chem Theory Comput. 2025 Oct 16;21(20):10515–26. doi: 10.1021/acs.jctc.5c00130 (PMC12573762; doi:10.1021/acs.jctc.5c00130)
Supplement: Supplementary file 1 [file ct5c00130_si_001.pdf]

Supplementary information for:
Exploring the Convergence and Properties of Intrinsic Bond Orbitals in Solids

Benjamin Wöckinger, Alexander Rumpf, and Tobias Schäfer*
Institute for Theoretical Physics, TU Wien, Wiedner Hauptstraße 8-10/136, A-1040 Vienna, Austria

ATOMIC STRUCTURES

Here we provide the considered atomic structures and cells of

- caffeine,
- benzene,
- coronene,
- graphene with flower defect,
- silicon with interstitial defect,

in the form of `POSCAR` files for VASP.

Caffeine

```
caffeine molecule
1.0
       19.0000000000   0.0000000000   0.0000000000
        0.0000000000  18.0000000000   0.0000000000
        0.0000000000   0.0000000000  12.0000000000
        O N C H
        2 4 8 10
Cartesian
   0.4700    2.5688    0.0006
  -3.1271   -0.4436   -0.0003
  -0.9686   -1.3125    0.0000
   2.2182    0.1412   -0.0003
  -1.3477    1.0797   -0.0001
   1.4119   -1.9372    0.0002
   0.8579    0.2592   -0.0008
   0.3897   -1.0264   -0.0004
   0.0307    1.4220   -0.0006
  -1.9061   -0.2495   -0.0004
   2.5032   -1.1998    0.0003
  -1.4276   -2.6960    0.0008
   3.1926    1.2061    0.0003
  -2.2969    2.1881    0.0007
   3.5163   -1.5787    0.0008
  -1.0451   -3.1973   -0.8937
  -2.5186   -2.7596    0.0011
  -1.0447   -3.1963    0.8957
   4.1992    0.7801    0.0002
   3.0468    1.8092   -0.8992
```

* tobias.schaefer@tuwien.ac.at

```
   3.0466    1.8083    0.9004
  -1.8087    3.1651   -0.0003
  -2.9322    2.1027    0.8881
  -2.9346    2.1021   -0.8849
```

Benzene

```
benzene molecule
1.0
  16.2000000000   0.0000000000   0.0000000000
   0.0000000000  16.1000000000   0.0000000000
   0.0000000000   0.0000000000  16.0000000000
    C    H
    6    6
Cart
 8.712645325   9.120995701   8.060540783
 9.258235731   7.840748099   8.124233518
 8.426884963   6.725473338   8.042650428
 7.050422155   6.889925942   7.899686405
 6.504474360   8.171050561   7.838453982
 7.336172399   9.286642887   7.916598567
 9.357841649   9.986399167   8.127737169
10.324954277   7.712900122   8.246743035
 8.850444649   5.731567320   8.094749952
 6.405544304   6.023726297   7.836286520
 5.436217209   8.299221149   7.726296885
 6.913099303  10.281000204   7.867113860
```

Coronene

```
coronene molecule
1.0
  20.21             0.0000000000   0.0000000000
   0.0000000000  20.21             0.0000000000
   0.0000000000   0.0000000000  14.458
    C    H
   24   12
Cart
 0.06546813853317   -1.23154341226572    1.72900013164781
 1.48753197971382   -1.23154341226572    1.72900013164781
 2.19856390030415    0.00000000000000    1.72900013164781
 1.48753197971382    1.23154341226572    1.72900013164781
 0.06546813853317    1.23154341226572    1.72900013164781
-0.64556378205716    0.00000000000000    1.72900013164781
 2.19529218179055   -2.45742004183783    1.72900013164781
 1.45962227520803   -3.67082275092130    1.72900013164781
 0.09337784303896   -3.67082275092130    1.72900013164781
-0.64229206354356   -2.45742004183783    1.72900013164781
-2.06108418621062    0.00000000000000    1.72900013164781
-2.74408680400649   -1.24381018244192    1.72900013164781
-2.06096458792196   -2.42701256847938    1.72900013164781
-2.74408680400649    1.24381018244192    1.72900013164781
-2.06096458792196    2.42701256847938    1.72900013164781
-0.64229206354356    2.45742004183783    1.72900013164781
 0.09337784303896    3.67082275092130    1.72900013164781
```

```
 1.45962227520803    3.67082275092130    1.72900013164781
 2.19529218179055    2.45742004183783    1.72900013164781
 3.61396470616894    2.42701256847938    1.72900013164781
 4.29708692225348    1.24381018244192    1.72900013164781
 3.61408430445760    0.00000000000000    1.72900013164781
 4.29708692225348   -1.24381018244192    1.72900013164781
 3.61396470616894   -2.42701256847938    1.72900013164781
 2.00258058359503   -4.60741684241786    1.72900013164781
-0.44958046534805   -4.60741684241786    1.72900013164781
-2.60059970999888   -3.36552530248663    1.72900013164781
-3.82668023447042   -1.24189153993123    1.72900013164781
-3.82668023447042    1.24189153993123    1.72900013164781
-2.60059970999888    3.36552530248663    1.72900013164781
-0.44958046534805    4.60741684241786    1.72900013164781
 2.00258058359503    4.60741684241786    1.72900013164781
 4.15359982824587    3.36552530248663    1.72900013164781
 5.37968035271741    1.24189153993123    1.72900013164781
 5.37968035271741   -1.24189153993123    1.72900013164781
 4.15359982824587   -3.36552530248663    1.72900013164781
```

Graphene flower defect

```
graphene flower defect
  1.0
    22.149000000  0.000000000  0.000000000
   -11.074500000 19.181596668  0.000000000
     0.000000000  0.000000000 14.000000000
  C
  162
  Direct
     0.957441843  0.015639991  0.500000000
     0.021364625  0.079562773  0.500000000
     0.021364625  0.144142492  0.500000000
     0.085114392  0.206615469  0.500000000
     0.943254924  0.199760769  0.500000000
     0.983649019  0.272959127  0.500000000
     0.947107998  0.311418992  0.500000000
     0.984257301  0.385664047  0.500000000
     0.947438566  0.422970210  0.500000000
     0.984436143  0.497175750  0.500000000
     0.947438566  0.534383713  0.500000000
     0.984257301  0.608508611  0.500000000
     0.947107998  0.645604364  0.500000000
     0.983649019  0.720605250  0.500000000
     0.943254924  0.753409513  0.500000000
     0.894138914  0.824664513  0.500000000
     0.892862124  0.887137490  0.500000000
     0.957441843  0.951717209  0.500000000
     0.021364625  0.951717209  0.500000000
     0.085287407  0.015639991  0.500000000
     0.085287407  0.079562773  0.500000000
     0.149867126  0.144142492  0.500000000
     0.148590336  0.206615469  0.500000000
     0.099474326  0.277870470  0.500000000
     0.059080231  0.310674733  0.500000000
     0.095621252  0.385675618  0.500000000
     0.058471949  0.422771371  0.500000000
```

0.095290684	0.496896269	0.500000000
0.058293107	0.534104232	0.500000000
0.095290684	0.608309772	0.500000000
0.058471949	0.645615935	0.500000000
0.095621252	0.719860991	0.500000000
0.059080231	0.758320855	0.500000000
0.099474326	0.831519213	0.500000000
0.957614858	0.824664513	0.500000000
0.021364625	0.887137490	0.500000000
0.085114392	0.888414280	0.500000000
0.148590336	0.951890224	0.500000000
0.149867126	0.015639991	0.500000000
0.212340103	0.079389758	0.500000000
0.212340103	0.142865702	0.500000000
0.205485403	0.277870470	0.500000000
0.172811607	0.318533955	0.500000000
0.208734293	0.390379327	0.500000000
0.169812852	0.424797347	0.500000000
0.206784260	0.498318887	0.500000000
0.169509588	0.534538478	0.500000000
0.206634669	0.608275013	0.500000000
0.169509588	0.644886466	0.500000000
0.206784260	0.718380731	0.500000000
0.169812852	0.754930862	0.500000000
0.208734293	0.828270323	0.500000000
0.172811607	0.864193009	0.500000000
0.205485403	0.937530290	0.500000000
0.278683761	0.977924385	0.500000000
0.316399367	0.053355597	0.500000000
0.283595104	0.093749692	0.500000000
0.324258589	0.167086973	0.500000000
0.283595104	0.199760769	0.500000000
0.316399367	0.272959127	0.500000000
0.278683761	0.310674733	0.500000000
0.317143626	0.385675618	0.500000000
0.282073754	0.424797347	0.500000000
0.318623886	0.498318887	0.500000000
0.280958778	0.534828298	0.500000000
0.317782651	0.608476043	0.500000000
0.280638437	0.645276897	0.500000000
0.317782651	0.719221965	0.500000000
0.280958778	0.756045838	0.500000000
0.318623886	0.830220356	0.500000000
0.282073754	0.867191765	0.500000000
0.317143626	0.941383364	0.500000000
0.391388681	0.978532667	0.500000000
0.428496005	0.052747315	0.500000000
0.391400252	0.089896618	0.500000000
0.430521981	0.164088218	0.500000000
0.396103961	0.203009659	0.500000000
0.430521981	0.276349120	0.500000000
0.391400252	0.311418992	0.500000000
0.428496005	0.385664047	0.500000000
0.391388681	0.422771371	0.500000000
0.428694844	0.496896269	0.500000000
0.392118150	0.534538478	0.500000000
0.428729603	0.608275013	0.500000000
0.391727719	0.645276897	0.500000000

0.428528573	0.719221965	0.500000000
0.391727719	0.756366179	0.500000000
0.428729603	0.830369947	0.500000000
0.392118150	0.867495028	0.500000000
0.428694844	0.941713932	0.500000000
0.502900384	0.978711509	0.500000000
0.539828866	0.052568473	0.500000000
0.502620903	0.089566050	0.500000000
0.540263112	0.163784954	0.500000000
0.504043521	0.201059626	0.500000000
0.540552932	0.275234144	0.500000000
0.504043521	0.312899252	0.500000000
0.540263112	0.386393516	0.500000000
0.502620903	0.422970210	0.500000000
0.539828866	0.497175750	0.500000000
0.502900384	0.534104232	0.500000000
0.540108347	0.608309772	0.500000000
0.502466138	0.644886466	0.500000000
0.538685729	0.718380731	0.500000000
0.502176318	0.756045838	0.500000000
0.538685729	0.830220356	0.500000000
0.502466138	0.867495028	0.500000000
0.540108347	0.941713932	0.500000000
0.614233245	0.978532667	0.500000000
0.651340569	0.052747315	0.500000000
0.614034406	0.089566050	0.500000000
0.650611100	0.163784954	0.500000000
0.613999647	0.200910035	0.500000000
0.651001531	0.274913803	0.500000000
0.614200677	0.312058017	0.500000000
0.651001531	0.386003085	0.500000000
0.613999647	0.423004969	0.500000000
0.650611100	0.496741504	0.500000000
0.614034406	0.534383713	0.500000000
0.651340569	0.608508611	0.500000000
0.614233245	0.645615935	0.500000000
0.651328998	0.719860991	0.500000000
0.612207270	0.754930862	0.500000000
0.646625289	0.828270323	0.500000000
0.612207270	0.867191765	0.500000000
0.651328998	0.941383364	0.500000000
0.726329884	0.977924385	0.500000000
0.764045489	0.053355597	0.500000000
0.725585625	0.089896618	0.500000000
0.760655496	0.164088218	0.500000000
0.724105365	0.201059626	0.500000000
0.761770472	0.275234144	0.500000000
0.724946599	0.312058017	0.500000000
0.762090813	0.386003085	0.500000000
0.724946599	0.422803939	0.500000000
0.761770472	0.496451684	0.500000000
0.724105365	0.532961095	0.500000000
0.760655496	0.606482635	0.500000000
0.725585625	0.645604364	0.500000000
0.764045489	0.720605250	0.500000000
0.726329884	0.758320855	0.500000000
0.759134147	0.831519213	0.500000000
0.718470661	0.864193009	0.500000000

```
    0.759134147  0.937530290  0.500000000
    0.894138914  0.079389758  0.500000000
    0.957614858  0.142865702  0.500000000
    0.837243847  0.093749692  0.500000000
    0.869917643  0.167086973  0.500000000
    0.833994957  0.203009659  0.500000000
    0.872916399  0.276349120  0.500000000
    0.835944990  0.312899252  0.500000000
    0.873219663  0.386393516  0.500000000
    0.836094581  0.423004969  0.500000000
    0.873219663  0.496741504  0.500000000
    0.835944990  0.532961095  0.500000000
    0.872916399  0.606482635  0.500000000
    0.833994957  0.640900655  0.500000000
    0.869917643  0.712746027  0.500000000
    0.837243847  0.753409513  0.500000000
    0.830389147  0.888414280  0.500000000
    0.830389147  0.951890224  0.500000000
    0.892862124  0.015639991  0.500000000
```

Silicon X interstitial

```
Silicon X interstitial (520 atoms)
1.0
      21.8788528442         0.0000000000         0.0000000000
       0.0000000000        21.8788528442         0.0000000000
       0.0000000000         0.0000000000        21.8788528442
   Si
  520
Direct
    0.101965073         0.022856597         0.027526432
    0.101965073         0.022856597         0.527526454
    0.101965073         0.522856605         0.027526432
    0.101965073         0.522856605         0.527526454
    0.601965073         0.022856597         0.027526432
    0.601965073         0.022856597         0.527526454
    0.601965073         0.522856605         0.027526432
    0.601965073         0.522856605         0.527526454
    0.022856597         0.101965073         0.027526432
    0.022856597         0.101965073         0.527526454
    0.022856597         0.601965073         0.027526432
    0.022856597         0.601965073         0.527526454
    0.522856605         0.101965073         0.027526432
    0.522856605         0.101965073         0.527526454
    0.522856605         0.601965073         0.027526432
    0.522856605         0.601965073         0.527526454
    0.995866032         0.995866032         0.996017547
    0.995866032         0.995866032         0.496017460
    0.995866032         0.495866076         0.996017547
    0.995866032         0.495866076         0.496017460
    0.495866076         0.995866032         0.996017547
    0.495866076         0.995866032         0.496017460
    0.495866076         0.495866076         0.996017547
    0.495866076         0.495866076         0.496017460
    0.250261664         0.998747605         0.000861127
    0.250261664         0.998747605         0.500861098
    0.250261664         0.498747605         0.000861127
```

0.250261664	0.498747605	0.500861098
0.750261708	0.998747605	0.000861127
0.750261708	0.998747605	0.500861098
0.750261708	0.498747605	0.000861127
0.750261708	0.498747605	0.500861098
0.998747605	0.250261664	0.000861127
0.998747605	0.250261664	0.500861098
0.998747605	0.750261708	0.000861127
0.998747605	0.750261708	0.500861098
0.498747605	0.250261664	0.000861127
0.498747605	0.250261664	0.500861098
0.498747605	0.750261708	0.000861127
0.498747605	0.750261708	0.500861098
0.249773512	0.249773512	0.999446073
0.249773512	0.249773512	0.499446160
0.249773512	0.749773512	0.999446073
0.249773512	0.749773512	0.499446160
0.749773512	0.249773512	0.999446073
0.749773512	0.249773512	0.499446160
0.749773512	0.749773512	0.999446073
0.749773512	0.749773512	0.499446160
0.999731841	0.999731841	0.250053876
0.999731841	0.999731841	0.750053876
0.999731841	0.499731798	0.250053876
0.999731841	0.499731798	0.750053876
0.499731798	0.999731841	0.250053876
0.499731798	0.999731841	0.750053876
0.499731798	0.499731798	0.250053876
0.499731798	0.499731798	0.750053876
0.249916397	0.999819716	0.249595888
0.249916397	0.999819716	0.749595844
0.249916397	0.499819760	0.249595888
0.249916397	0.499819760	0.749595844
0.749916397	0.999819716	0.249595888
0.749916397	0.999819716	0.749595844
0.749916397	0.499819760	0.249595888
0.749916397	0.499819760	0.749595844
0.999819716	0.249916397	0.249595888
0.999819716	0.249916397	0.749595844
0.999819716	0.749916397	0.249595888
0.999819716	0.749916397	0.749595844
0.499819760	0.249916397	0.249595888
0.499819760	0.249916397	0.749595844
0.499819760	0.749916397	0.249595888
0.499819760	0.749916397	0.749595844
0.249601772	0.249601772	0.250873303
0.249601772	0.249601772	0.750873346
0.249601772	0.749601772	0.250873303
0.249601772	0.749601772	0.750873346
0.749601772	0.249601772	0.250873303
0.749601772	0.249601772	0.750873346
0.749601772	0.749601772	0.250873303
0.749601772	0.749601772	0.750873346
0.128970455	0.128970455	0.996066454
0.128970455	0.128970455	0.496066454
0.128970455	0.628970466	0.996066454
0.128970455	0.628970466	0.496066454
0.628970466	0.128970455	0.996066454

0.628970466	0.128970455	0.496066454
0.628970466	0.628970466	0.996066454
0.628970466	0.628970466	0.496066454
0.374691565	0.126273710	0.000893315
0.374691565	0.126273710	0.500893310
0.374691565	0.626273710	0.000893315
0.374691565	0.626273710	0.500893310
0.874691522	0.126273710	0.000893315
0.874691522	0.126273710	0.500893310
0.874691522	0.626273710	0.000893315
0.874691522	0.626273710	0.500893310
0.126273710	0.374691565	0.000893315
0.126273710	0.374691565	0.500893310
0.126273710	0.874691522	0.000893315
0.126273710	0.874691522	0.500893310
0.626273710	0.374691565	0.000893315
0.626273710	0.374691565	0.500893310
0.626273710	0.874691522	0.000893315
0.626273710	0.874691522	0.500893310
0.375103262	0.375103262	0.999455139
0.375103262	0.375103262	0.499455226
0.375103262	0.875103262	0.999455139
0.375103262	0.875103262	0.499455226
0.875103262	0.375103262	0.999455139
0.875103262	0.375103262	0.499455226
0.875103262	0.875103262	0.999455139
0.875103262	0.875103262	0.499455226
0.125214533	0.125214533	0.250042739
0.125214533	0.125214533	0.750042717
0.125214533	0.625214501	0.250042739
0.125214533	0.625214501	0.750042717
0.625214501	0.125214533	0.250042739
0.625214501	0.125214533	0.750042717
0.625214501	0.625214501	0.250042739
0.625214501	0.625214501	0.750042717
0.374968878	0.125094337	0.249609880
0.374968878	0.125094337	0.749609880
0.374968878	0.625094370	0.249609880
0.374968878	0.625094370	0.749609880
0.874968921	0.125094337	0.249609880
0.874968921	0.125094337	0.749609880
0.874968921	0.625094370	0.249609880
0.874968921	0.625094370	0.749609880
0.125094337	0.374968878	0.249609880
0.125094337	0.374968878	0.749609880
0.125094337	0.874968921	0.249609880
0.125094337	0.874968921	0.749609880
0.625094370	0.374968878	0.249609880
0.625094370	0.374968878	0.749609880
0.625094370	0.874968921	0.249609880
0.625094370	0.874968921	0.749609880
0.375383408	0.375383408	0.250858744
0.375383408	0.375383408	0.750858788
0.375383408	0.875383364	0.250858744
0.375383408	0.875383364	0.750858788
0.875383364	0.375383408	0.250858744
0.875383364	0.375383408	0.750858788
0.875383364	0.875383364	0.250858744

0.875383364	0.875383364	0.750858788
0.996615499	0.128242085	0.127911409
0.996615499	0.128242085	0.627911387
0.996615499	0.628242096	0.127911409
0.996615499	0.628242096	0.627911387
0.496615543	0.128242085	0.127911409
0.496615543	0.128242085	0.627911387
0.496615543	0.628242096	0.127911409
0.496615543	0.628242096	0.627911387
0.250542115	0.124734053	0.125272289
0.250542115	0.124734053	0.625272256
0.250542115	0.624734064	0.125272289
0.250542115	0.624734064	0.625272256
0.750542071	0.124734053	0.125272289
0.750542071	0.124734053	0.625272256
0.750542071	0.624734064	0.125272289
0.750542071	0.624734064	0.625272256
0.000194865	0.374353621	0.125257240
0.000194865	0.374353621	0.625257261
0.000194865	0.874353621	0.125257240
0.000194865	0.874353621	0.625257261
0.500194842	0.374353621	0.125257240
0.500194842	0.374353621	0.625257261
0.500194842	0.874353621	0.125257240
0.500194842	0.874353621	0.625257261
0.252227936	0.372697941	0.125980597
0.252227936	0.372697941	0.625980619
0.252227936	0.872697941	0.125980597
0.252227936	0.872697941	0.625980619
0.752227914	0.372697941	0.125980597
0.752227914	0.372697941	0.625980619
0.752227914	0.872697941	0.125980597
0.752227914	0.872697941	0.625980619
0.000023466	0.124920287	0.374031673
0.000023466	0.124920287	0.874031673
0.000023466	0.624920320	0.374031673
0.000023466	0.624920320	0.874031673
0.500023494	0.124920287	0.374031673
0.500023494	0.124920287	0.874031673
0.500023494	0.624920320	0.374031673
0.500023494	0.624920320	0.874031673
0.250766227	0.124541423	0.374286930
0.250766227	0.124541423	0.874286930
0.250766227	0.624541402	0.374286930
0.250766227	0.624541402	0.874286930
0.750766205	0.124541423	0.374286930
0.750766205	0.124541423	0.874286930
0.750766205	0.624541402	0.374286930
0.750766205	0.624541402	0.874286930
0.000415239	0.374151281	0.374307242
0.000415239	0.374151281	0.874307242
0.000415239	0.874151281	0.374307242
0.000415239	0.874151281	0.874307242
0.500415271	0.374151281	0.374307242
0.500415271	0.374151281	0.874307242
0.500415271	0.874151281	0.374307242
0.500415271	0.874151281	0.874307242
0.249964889	0.374980472	0.375186517

0.249964889	0.374980472	0.875186604
0.249964889	0.874980429	0.375186517
0.249964889	0.874980429	0.875186604
0.749964867	0.374980472	0.375186517
0.749964867	0.374980472	0.875186604
0.749964867	0.874980429	0.375186517
0.749964867	0.874980429	0.875186604
0.128242085	0.996615499	0.127911409
0.128242085	0.996615499	0.627911387
0.128242085	0.496615543	0.127911409
0.128242085	0.496615543	0.627911387
0.628242096	0.996615499	0.127911409
0.628242096	0.996615499	0.627911387
0.628242096	0.496615543	0.127911409
0.628242096	0.496615543	0.627911387
0.374353621	0.000194865	0.125257240
0.374353621	0.000194865	0.625257261
0.374353621	0.500194842	0.125257240
0.374353621	0.500194842	0.625257261
0.874353621	0.000194865	0.125257240
0.874353621	0.000194865	0.625257261
0.874353621	0.500194842	0.125257240
0.874353621	0.500194842	0.625257261
0.124734053	0.250542115	0.125272289
0.124734053	0.250542115	0.625272256
0.124734053	0.750542071	0.125272289
0.124734053	0.750542071	0.625272256
0.624734064	0.250542115	0.125272289
0.624734064	0.250542115	0.625272256
0.624734064	0.750542071	0.125272289
0.624734064	0.750542071	0.625272256
0.372697941	0.252227936	0.125980597
0.372697941	0.252227936	0.625980619
0.372697941	0.752227914	0.125980597
0.372697941	0.752227914	0.625980619
0.872697941	0.252227936	0.125980597
0.872697941	0.252227936	0.625980619
0.872697941	0.752227914	0.125980597
0.872697941	0.752227914	0.625980619
0.124920287	0.000023466	0.374031673
0.124920287	0.000023466	0.874031673
0.124920287	0.500023494	0.374031673
0.124920287	0.500023494	0.874031673
0.624920320	0.000023466	0.374031673
0.624920320	0.000023466	0.874031673
0.624920320	0.500023494	0.374031673
0.624920320	0.500023494	0.874031673
0.374151281	0.000415239	0.374307242
0.374151281	0.000415239	0.874307242
0.374151281	0.500415271	0.374307242
0.374151281	0.500415271	0.874307242
0.874151281	0.000415239	0.374307242
0.874151281	0.000415239	0.874307242
0.874151281	0.500415271	0.374307242
0.874151281	0.500415271	0.874307242
0.124541423	0.250766227	0.374286930
0.124541423	0.250766227	0.874286930
0.124541423	0.750766205	0.374286930

0.124541423	0.750766205	0.874286930
0.624541402	0.250766227	0.374286930
0.624541402	0.250766227	0.874286930
0.624541402	0.750766205	0.374286930
0.624541402	0.750766205	0.874286930
0.374980472	0.249964889	0.375186517
0.374980472	0.249964889	0.875186604
0.374980472	0.749964867	0.375186517
0.374980472	0.749964867	0.875186604
0.874980429	0.249964889	0.375186517
0.874980429	0.249964889	0.875186604
0.874980429	0.749964867	0.375186517
0.874980429	0.749964867	0.875186604
0.312471602	0.062497821	0.062036699
0.312471602	0.062497821	0.562036672
0.312471602	0.562497799	0.062036699
0.312471602	0.562497799	0.562036672
0.812471645	0.062497821	0.062036699
0.812471645	0.062497821	0.562036672
0.812471645	0.562497799	0.062036699
0.812471645	0.562497799	0.562036672
0.062497821	0.312471602	0.062036699
0.062497821	0.312471602	0.562036672
0.062497821	0.812471645	0.062036699
0.062497821	0.812471645	0.562036672
0.562497799	0.312471602	0.062036699
0.562497799	0.312471602	0.562036672
0.562497799	0.812471645	0.062036699
0.562497799	0.812471645	0.562036672
0.312434486	0.312434486	0.061682208
0.312434486	0.312434486	0.561682208
0.312434486	0.812434508	0.061682208
0.312434486	0.812434508	0.561682208
0.812434508	0.312434486	0.061682208
0.812434508	0.312434486	0.561682208
0.812434508	0.812434508	0.061682208
0.812434508	0.812434508	0.561682208
0.062472528	0.062472528	0.311603464
0.062472528	0.062472528	0.811603442
0.062472528	0.562472517	0.311603464
0.062472528	0.562472517	0.811603442
0.562472517	0.062472528	0.311603464
0.562472517	0.062472528	0.811603442
0.562472517	0.562472517	0.311603464
0.562472517	0.562472517	0.811603442
0.312437733	0.062452461	0.311262403
0.312437733	0.062452461	0.811262403
0.312437733	0.562452423	0.311262403
0.312437733	0.562452423	0.811262403
0.812437733	0.062452461	0.311262403
0.812437733	0.062452461	0.811262403
0.812437733	0.562452423	0.311262403
0.812437733	0.562452423	0.811262403
0.062452461	0.312437733	0.311262403
0.062452461	0.312437733	0.811262403
0.062452461	0.812437733	0.311262403
0.062452461	0.812437733	0.811262403
0.562452423	0.312437733	0.311262403

0.562452423	0.312437733	0.811262403
0.562452423	0.812437733	0.311262403
0.562452423	0.812437733	0.811262403
0.312497886	0.312497886	0.312714021
0.312497886	0.312497886	0.812714000
0.312497886	0.812497886	0.312714021
0.312497886	0.812497886	0.812714000
0.812497886	0.312497886	0.312714021
0.812497886	0.312497886	0.812714000
0.812497886	0.812497886	0.312714021
0.812497886	0.812497886	0.812714000
0.188178308	0.188178308	0.064115376
0.188178308	0.188178308	0.564115425
0.188178308	0.688178308	0.064115376
0.188178308	0.688178308	0.564115425
0.688178308	0.188178308	0.064115376
0.688178308	0.188178308	0.564115425
0.688178308	0.688178308	0.064115376
0.688178308	0.688178308	0.564115425
0.434546005	0.190447871	0.064981503
0.434546005	0.190447871	0.564981536
0.434546005	0.690447849	0.064981503
0.434546005	0.690447849	0.564981536
0.934546005	0.190447871	0.064981503
0.934546005	0.190447871	0.564981536
0.934546005	0.690447849	0.064981503
0.934546005	0.690447849	0.564981536
0.190447871	0.434546005	0.064981503
0.190447871	0.434546005	0.564981536
0.190447871	0.934546005	0.064981503
0.190447871	0.934546005	0.564981536
0.690447849	0.434546005	0.064981503
0.690447849	0.434546005	0.564981536
0.690447849	0.934546005	0.064981503
0.690447849	0.934546005	0.564981536
0.436692233	0.436692233	0.064126284
0.436692233	0.436692233	0.564126235
0.436692233	0.936692233	0.064126284
0.436692233	0.936692233	0.564126235
0.936692233	0.436692233	0.064126284
0.936692233	0.436692233	0.564126235
0.936692233	0.936692233	0.064126284
0.936692233	0.936692233	0.564126235
0.187339331	0.187339331	0.313190666
0.187339331	0.187339331	0.813190687
0.187339331	0.687339310	0.313190666
0.187339331	0.687339310	0.813190687
0.687339310	0.187339331	0.313190666
0.687339310	0.187339331	0.813190687
0.687339310	0.687339310	0.313190666
0.687339310	0.687339310	0.813190687
0.437344889	0.187558954	0.312108855
0.437344889	0.187558954	0.812108899
0.437344889	0.687558954	0.312108855
0.437344889	0.687558954	0.812108899
0.937344933	0.187558954	0.312108855
0.937344933	0.187558954	0.812108899
0.937344933	0.687558954	0.312108855

0.937344933	0.687558954	0.812108899
0.187558954	0.437344889	0.312108855
0.187558954	0.437344889	0.812108899
0.187558954	0.937344933	0.312108855
0.187558954	0.937344933	0.812108899
0.687558954	0.437344889	0.312108855
0.687558954	0.437344889	0.812108899
0.687558954	0.937344933	0.312108855
0.687558954	0.937344933	0.812108899
0.437611391	0.437611391	0.313231683
0.437611391	0.437611391	0.813231748
0.437611391	0.937611435	0.313231683
0.437611391	0.937611435	0.813231748
0.937611435	0.437611391	0.313231683
0.937611435	0.437611391	0.813231748
0.937611435	0.937611435	0.313231683
0.937611435	0.937611435	0.813231748
0.063016087	0.187850498	0.187822536
0.063016087	0.187850498	0.687822536
0.063016087	0.687850476	0.187822536
0.063016087	0.687850476	0.687822536
0.563016070	0.187850498	0.187822536
0.563016070	0.187850498	0.687822536
0.563016070	0.687850476	0.187822536
0.563016070	0.687850476	0.687822536
0.312011064	0.187853593	0.187964309
0.312011064	0.187853593	0.687964330
0.312011064	0.687853571	0.187964309
0.312011064	0.687853571	0.687964330
0.812011086	0.187853593	0.187964309
0.812011086	0.187853593	0.687964330
0.812011086	0.687853571	0.187964309
0.812011086	0.687853571	0.687964330
0.061884247	0.437020065	0.187859041
0.061884247	0.437020065	0.687859063
0.061884247	0.937020021	0.187859041
0.061884247	0.937020021	0.687859063
0.561884242	0.437020065	0.187859041
0.561884242	0.437020065	0.687859063
0.561884242	0.937020021	0.187859041
0.561884242	0.937020021	0.687859063
0.312906466	0.437078169	0.187950338
0.312906466	0.437078169	0.687950295
0.312906466	0.937078082	0.187950338
0.312906466	0.937078082	0.687950295
0.812906488	0.437078169	0.187950338
0.812906488	0.437078169	0.687950295
0.812906488	0.937078082	0.187950338
0.812906488	0.937078082	0.687950295
0.060695154	0.188620517	0.436959607
0.060695154	0.188620517	0.936959607
0.060695154	0.688620517	0.436959607
0.060695154	0.688620517	0.936959607
0.560695181	0.188620517	0.436959607
0.560695181	0.188620517	0.936959607
0.560695181	0.688620517	0.436959607
0.560695181	0.688620517	0.936959607
0.312259673	0.187267235	0.437182346

0.312259673	0.187267235	0.937182346
0.312259673	0.687267257	0.437182346
0.312259673	0.687267257	0.937182346
0.812259716	0.187267235	0.437182346
0.812259716	0.187267235	0.937182346
0.812259716	0.687267257	0.437182346
0.812259716	0.687267257	0.937182346
0.064288969	0.436303028	0.436972030
0.064288969	0.436303028	0.936971986
0.064288969	0.936302984	0.436972030
0.064288969	0.936302984	0.936971986
0.564288952	0.436303028	0.436972030
0.564288952	0.436303028	0.936971986
0.564288952	0.936302984	0.436972030
0.564288952	0.936302984	0.936971986
0.312683204	0.437666967	0.437191064
0.312683204	0.437666967	0.937191064
0.312683204	0.937667054	0.437191064
0.312683204	0.937667054	0.937191064
0.812683226	0.437666967	0.437191064
0.812683226	0.437666967	0.937191064
0.812683226	0.937667054	0.437191064
0.812683226	0.937667054	0.937191064
0.187850498	0.063016087	0.187822536
0.187850498	0.063016087	0.687822536
0.187850498	0.563016070	0.187822536
0.187850498	0.563016070	0.687822536
0.687850476	0.063016087	0.187822536
0.687850476	0.063016087	0.687822536
0.687850476	0.563016070	0.187822536
0.687850476	0.563016070	0.687822536
0.437020065	0.061884247	0.187859041
0.437020065	0.061884247	0.687859063
0.437020065	0.561884242	0.187859041
0.437020065	0.561884242	0.687859063
0.937020021	0.061884247	0.187859041
0.937020021	0.061884247	0.687859063
0.937020021	0.561884242	0.187859041
0.937020021	0.561884242	0.687859063
0.187853593	0.312011064	0.187964309
0.187853593	0.312011064	0.687964330
0.187853593	0.812011086	0.187964309
0.187853593	0.812011086	0.687964330
0.687853571	0.312011064	0.187964309
0.687853571	0.312011064	0.687964330
0.687853571	0.812011086	0.187964309
0.687853571	0.812011086	0.687964330
0.437078169	0.312906466	0.187950338
0.437078169	0.312906466	0.687950295
0.437078169	0.812906488	0.187950338
0.437078169	0.812906488	0.687950295
0.937078082	0.312906466	0.187950338
0.937078082	0.312906466	0.687950295
0.937078082	0.812906488	0.187950338
0.937078082	0.812906488	0.687950295
0.188620517	0.060695154	0.436959607
0.188620517	0.060695154	0.936959607
0.188620517	0.560695181	0.436959607

0.188620517	0.560695181	0.936959607
0.688620517	0.060695154	0.436959607
0.688620517	0.060695154	0.936959607
0.688620517	0.560695181	0.436959607
0.688620517	0.560695181	0.936959607
0.436303028	0.064288969	0.436972030
0.436303028	0.064288969	0.936971986
0.436303028	0.564288952	0.436972030
0.436303028	0.564288952	0.936971986
0.936302984	0.064288969	0.436972030
0.936302984	0.064288969	0.936971986
0.936302984	0.564288952	0.436972030
0.936302984	0.564288952	0.936971986
0.187267235	0.312259673	0.437182346
0.187267235	0.312259673	0.937182346
0.187267235	0.812259716	0.437182346
0.187267235	0.812259716	0.937182346
0.687267257	0.312259673	0.437182346
0.687267257	0.312259673	0.937182346
0.687267257	0.812259716	0.437182346
0.687267257	0.812259716	0.937182346
0.437666967	0.312683204	0.437191064
0.437666967	0.312683204	0.937191064
0.437666967	0.812683226	0.437191064
0.437666967	0.812683226	0.937191064
0.937667054	0.312683204	0.437191064
0.937667054	0.312683204	0.937191064
0.937667054	0.812683226	0.437191064
0.937667054	0.812683226	0.937191064
